# Enhancing Human–Robot Collaboration through a Multi-Module Interaction Framework with Sensor Fusion: Object Recognition, Verbal Communication, User of Interest Detection, Gesture and Gaze Recognition

**DOI:** 10.3390/s23135798

**Published:** 2023-06-21

**Authors:** Shuvo Kumar Paul, Mircea Nicolescu, Monica Nicolescu

**Affiliations:** Department of Computer Science and Engineering, University of Nevada, Reno, 1664 N Virginia St, Reno, NV 89557, USA; mircea@cse.unr.edu (M.N.); monica@cse.unr.edu (M.N.)

**Keywords:** HRI, human–AI interaction, interaction interface, gaze estimation, pose estimation, multi-modal inputs, multi-party interaction, collaborative HRI, gesture recognition, information extraction

## Abstract

With the increasing presence of robots in our daily lives, it is crucial to design interaction interfaces that are natural, easy to use and meaningful for robotic tasks. This is important not only to enhance the user experience but also to increase the task reliability by providing supplementary information. Motivated by this, we propose a multi-modal framework consisting of multiple independent modules. These modules take advantage of multiple sensors (e.g., image, sound, depth) and can be used separately or in combination for effective human–robot collaborative interaction. We identified and implemented four key components of an effective human robot collaborative setting, which included determining object location and pose, extracting intricate information from verbal instructions, resolving user(s) of interest (UOI), and gesture recognition and gaze estimation to facilitate the natural and intuitive interactions. The system uses a feature–detector–descriptor approach for object recognition and a homography-based technique for planar pose estimation and a deep multi-task learning model to extract intricate task parameters from verbal communication. The user of interest (UOI) is detected by estimating the facing state and active speakers. The framework also includes gesture detection and gaze estimation modules, which are combined with a verbal instruction component to form structured commands for robotic entities. Experiments were conducted to assess the performance of these interaction interfaces, and the results demonstrated the effectiveness of the approach.

## 1. Introduction

The robotics revolution is driven by advancements in automation, engineering and AI, leading to the integration of robots into daily life. Unlike industrial robots, service robots engage in diverse tasks while interacting with humans. To ensure seamless integration, intuitive interaction interfaces and user trust are crucial. This requires a human–robot interaction (HRI) framework that facilitates natural interaction and enhances task reliability with multi-modal sensing and sensor fusion technologies. By combining visual, auditory, depth sensors, etc., robots can achieve a comprehensive understanding of their environment and human partners, and this can aid in clarifying or deducing absent task parameters, thereby enhancing their reliability in performing designated tasks.

As robotic technology continues to advance, these entities will become an integral part of modern society and have the potential to influence our social experiences. However, their successful integration will heavily depend on whether or not users trust robots to accurately complete tasks. Therefore, it is essential to ensure that robots can consistently execute tasks in a proper and reliable manner to build and maintain this trust. In order to facilitate efficient human–robot interaction within a collaborative setting involving multiple users and object manipulation, we suggest the four following key components: (1) recognizing objects within the scene; (2) obtaining clear task parameters; (3) identifying the intended user(s) or user(s) of interest; and (4) incorporating gestures and gaze to facilitate natural interaction and provide supplementary task-related information. By integrating these four components, we can ensure a clear understanding of tasks, including which objects to manipulate and what actions to perform. This approach enables targeted user interaction and facilitates natural communication, ultimately enhancing the overall effectiveness and success of human–robot collaboration.

Additionally, to properly execute robotic tasks, instructions must include a set of parameters that define a task configuration. We propose the two essential properties of a **complete** task configuration:

1. To execute the intended task, the task configuration should comprise all the essential task parameter details. The necessary task parameters can vary depending on the task, such as the navigational task, which may require direction, and an assisting task, which may need information related to objects, their attributes, locations in the environment and the order of task initiation. The robot can then accurately interpret structured instructions by extracting task parameters from its sensors and filtering them.
2. The reliability of task configurations is essential for the successful execution of intended tasks by robots. Even when the robot adheres to the given instructions, errors in the task parameters can still occur due to the noisy sensory data or ambiguities during parameter extraction. For instance, a robot may turn right instead of executing a rightward movement. To mitigate this issue, the cross-validation of instructions is necessary to establish the user’s intent. This can be achieved by integrating sensory information with a range of interfaces designed for human–robot interaction.

The initial statement asserts that the first property alone can be adequate for executing a robotic task, but the second property offers greater certainty about the validity of task configurations. Thus, if sensor input streams cannot determine the necessary task parameters, interaction interfaces can be used to establish and confirm these parameters, and vice versa. Although attaining the second property may be unfeasible for every task configuration, verifying instructions from multiple sensor streams and interaction interfaces should be a goal in collaborative human–robot interaction (HRI) design, as it would lead to more dependable task configurations. More dependable task configurations would contribute to successful task completion and strengthen trust in robots. These factors necessitate the use of natural interaction interfaces.

In natural human interactions, different modalities are used to effectively convey information. These modalities include gestures, speech and facial expressions, among others. Two very important natural interaction interfaces for humans are gaze [1] and gestures [2], particularly pointing gestures, which can convey simple messages or commands to the robot. Although they are inadequate for conveying complex information, these interfaces provide a more intuitive and natural way of communicating simpler instructions in noisy environments.

Moreover, these interaction interfaces allow for the intuitive specification of objects and their locations and can serve as straightforward yet meaningful commands. Additionally, particular human gestures and gaze indicate specific information that can convey the user’s general intent. This inferred intent information can then be compared or matched with predefined gesture and gaze configurations to provide supplementary information or appropriate commands for the robot to execute specific tasks.

Speech is a natural means of conveying complex commands that can be effectively parsed and processed by modern natural language processing (NLP) techniques. Using natural language allows for faster and more intrinsic communication between humans and robots. However, natural language commands must first be translated into a formal language that the robot can understand and act upon. The robot then formulates a structured message that is transformed back into natural language for easier comprehension by the user.

Additionally, for natural and effective verbal and non-verbal interactions, it is essential to understand the roles, i.e., speaker, addressee and bystander known as footing [3]. For a robot to participate seamlessly in multi-party conversations and interactions, it must be able to accurately infer these roles.

While humans can detect active speakers and assume their roles during conversations [4], robots find it challenging to perform such precise interpretation as the task of active speaker detection is inherently multi-modal, requiring the synthesis and comprehension of audio-visual and sometimes linguistic information. It is also necessary for the robot to infer whether the instructions were intended for it or not. While this scenario is relatively simple in a single-user environment, it becomes much more complex in a multi-user collaborative settings where users can communicate and direct instructions to one another and the robot. Thus, in a multi-party collaborative human–robot interaction (HRI) settings, the robot must reliably determine whether it is being addressed and identify the user of interest (UOI) who is directing the instructions.

Once we established the validity of the task parameters, we need to provide a robot with reliable information of the object(s) of interest (OOI) in the 3D space so that it can effectively manipulate the object. Object detection and respective pose estimation are two critical components that help robots better understand and interact with their environment. Object detection allows robots to identify and locate objects in their field of view, enabling them to make informed decisions based on their surroundings. Pose estimation, on the other hand, gives robots the ability to determine the 3D position and orientation of objects in space, a crucial piece of information for tasks such as object grasping and obstacle avoidance. The combination of object detection and pose estimation provides robots with a comprehensive understanding of their environment, enabling them to perform tasks more effectively and efficiently.

We propose a simple and reliable HRI framework that addresses the challenges mentioned to date. The components within our framework offer flexibility, as they can be utilized either individually or combined with other modules based on the requirements of the specific interaction scenario. To this effect, our contributions are as follows:

1. We use a feature-detector-descriptor-based method for detection and a homography-based pose estimation technique where, by utilizing the fusion of image and depth information, we estimate the pose of an object in terms of a 2D planar representation in 3D space.
2. An NLP system that extracts a set of information from verbal instructions retrieved from the audio input; verbal instructions with robot are parsed to extract task action, object of interest and its attributes and position in the 2D image frame.
3. Detect the user(s) of interest (UOI(s)):
  - Detecting the facing state—determining whether the user is facing the robot’s viewpoint camera or not.
  - Detecting the speaking state—identifying whether the user is speaking or not.
4. A gesture recognition system that estimates whether the user is performing a pointing gesture and the pointing direction.
5. A gaze estimation technique using only 2D images that can be used to direct the attention of the interacting robots towards an object or a certain location in the scene.
6. Finally, we show how pointing gesture recognition and gaze estimation can be used in conjunction with the information extracted from the verbal instruction and the detected objects in the scene to generate reliable robotic task configurations by disambiguating and supplementing the necessary task parameters.

The proposed system has the potential to greatly enhance the interaction between humans and robots. The integration of one or multiple of these interaction interfaces can allow for a more comprehensive understanding of the environment and user inputs, leading to improved task execution, increased reliability and an overall enhanced user experience.

In the subsequent section, a brief overview of the prior work is provided. Next, we elaborate on the methodology of each module in detail. Subsequently, the following chapters present our evaluation, encompassing experimental outcomes and observations. Finally, we conclude this paper by summarizing our work and suggesting future directions for further investigation.

## 2. Related Work

The following subsections will cover some previous research related to proposed modules.

### 2.1. Object Detection

The detection of objects remains a central challenge in the field of computer vision, and the introduction of feature detectors and descriptors has been recognized as a significant advancement in this area. The academic literature has seen the emergence of a wide range of detectors, descriptors and their various adaptations in recent decades. The applications of these methods have been extended to several other vision-based domains, including but not limited to panorama stitching, tracking and visual navigation.

The Harris corner detector, which has been widely acknowledged as one of the earliest feature detectors, was originally introduced by Harris et al. [5]. Subsequently, Tomasi et al. [6] developed the Kanade–Lucas–Tomasi (KLT) tracker, building upon the Harris corner detector. The Good Features To Track (GFTT) detection metric was proposed by Shi and Tomasi [7], who demonstrated its superior performance compared to existing techniques. Hall et al. [8] introduced the concept of saliency with regard to scale change, and evaluated the Harris method proposed in [9] as well as the Harris Laplacian corner detector [10], which combines the Harris detector with the Laplacian function.

Lowe’s 2004 paper on scale invariant feature transform (SIFT) is a landmark contribution to the field of computer vision, driven by the need for a feature detector that can operate independently of the image scale. SIFT serves both as a detector and descriptor of image features. In 2008, H. Bay et al. introduced speeded up robust features (SURF) as an alternative to SIFT, but both approaches require significant computational resources. The SIFT detector employs the difference of Gaussians (DoG) at varying scales, whereas the SURF detector uses a Haar wavelet approximation of the determinant of the Hessian matrix to expedite the detection process. Numerous versions of SIFT [11,12,13,14] and SURF [15,16,17] have been presented in the past, with the aim of addressing various issues and reporting improvements in matching. Nevertheless, the issue of execution time continues to pose a challenge for numerous vision applications.

Various detectors have been developed to improve execution time in computer vision applications. For example, FAST [18] and AGAST [19] are among the detectors developed to enhance the performance. Calonder et al. developed the BRIEF [20] descriptor, which utilizes binary strings and offers an efficient processing time. Another descriptor, ORB [21] was presented by Rublee et al. which combines the modified FAST for feature detection with BRIEF for description. BRISK [22] employs AGAST and FAST for corner detection and filtering, respectively. In contrast, the FREAK [23] method utilizes a circular sampling grid to generate retinal sampling patterns and constructs a binary descriptor through the application of a one-bit difference of Gaussians (DoG) technique. The KAZE and AKAZE methods, introduced by Alcantarilla et al., utilize non-linear scale-space through non-linear diffusion filtering, with the latter utilizing a more computationally efficient method called fast explicit diffusion (FED) [24,25].

In our work, we have selected four methods to investigate: SIFT, SURF, FAST+BRISK and AKAZE. We selected these descriptors based on the comprehensive comparisons conducted in recent literature [26,27,28]. To achieve an optimal solution, we carefully chose two floating-point detector-descriptors (SIFT and SURF) and two binary detector-descriptors (AKAZE and BRISK). Floating point-based detector-descriptors are known for their accuracy, while the binary detector-descriptors offer faster processing speeds. Our goal was to strike a balance and find a solution that delivers the best possible results.

### 2.2. Planar Pose Estimation

Planar pose estimation techniques have gained popularity in recent years across various fields, including robotics and augmented reality.

One technique proposed by Simon et al. [29] uses homography projection and consecutive image analysis to estimate the pose of planar structures. Changhai et al. [30] presents a robust method for estimating 3D poses of planes using weighted incremental normal estimation and Bayesian inference. Donoser et al. [31] utilized the properties of maximally stable extremal regions (MSERs [32]) to construct a perspectively invariant frame and estimate the planar pose. In our approach, we estimate the basis vectors of the object surface by applying perspective transformation to a set of corresponding points on the test image, and use depth information to compute the normal and estimate the 3D pose of the planar object.

### 2.3. Pointing Gesture Recognition

In the past, pointing gesture interfaces were predominantly developed with wearable devices, such as glove-based systems, as presented in [33,34]. To locate pointed objects by interpreting the pointing gestures, Kahn et al. introduced the Perseus architecture [35,36] that utilized several feature maps, including intensity, edge, motion, disparity and color. Kadobayashi et al. proposed a gesture interpreter termed VisTA-Walk which employed the Pfinder algorithm [37], a multi-class statistical color and shape model that can extract the 2D representations of the head and hands under different viewing conditions. More recently, researchers have explored different approaches to solve the pointing gesture detection problem, including using stereo cameras, depth cameras or multi-cameras [38,39,40].

The utilization of hidden Markov models (HMMs) in detecting pointing gestures has been extensively explored in the literature. Wilson et al. [41] proposed a parametric HMM, which allows for the recognition, representation and interpretation of parameterized gestures such as pointing. Nickel et al. [42] integrated dense disparity maps of a person’s face and hands with a hidden Markov model (HMM) in order to detect pointing gestures. Park et al. [43] applied the cascade HMM with particle filters that requires a significant number of HMM states for precise gesture recognition; however, it results in prolonged processing times.

In their comprehensive review of hand gesture recognition, Rautaray et al. [44] identified the recognized constraints associated with the popular approaches in this field.

This study utilizes the estimated (image) coordinates of the user’s forearm joints, namely the elbow and wrist, to achieve two objectives. Firstly, to distinguish whether the user is executing pointing gestures; and secondly, to deduce the general direction in which the user is pointing, such as left, right or straight. To accomplish the latter, the system computes the line that intersects the arm joints and subsequently utilizes this information to estimate the pointing direction.

### 2.4. Natural Language Understanding in HRI

Natural language has been widely studied as a means of interaction between humans and robots in various contexts such as navigation, manipulation and task execution. The use of natural language understanding (NLU) has been employed along with other sensory information, such as vision, to improve the interpretation of human instructions or scene configurations. The primary objective of NLU in human–robot interaction (HRI) can be broadly classified into two categories.

Numerous methods have been proposed by researchers to address the challenge of natural language-based interaction in human–robot interaction (HRI). One approach, developed by Kollar et al. [45], involves inferring the most probable path for an agent by extracting specific parameters from the verbal information. MacMahon et al. [46] introduced MARCO, an agent that deduces implied actions by combining linguistic conditional phrases, spatial action data and environmental arrangement. Statistical machine translation techniques were explored by Matuszek et al. [47] for following natural language route instructions.

Furthermore, some scientists suggested the implementation of robotic architectures that possess the capability to convert natural language commands into logical action language and goal representation. For instance, Cantrell et al. [48] designed a robotic architecture that features a planner that employs discovered knowledge to learn the previous and post-conditions of prior action sequences from natural language expressions. Additionally, Dzifcak et al. [49] have proposed an incremental process that converts natural language instructions into action language and goal representation. This representation can be analyzed to assess the feasibility of the objective and establish new action scripts designed to achieve the established goals.

Finally, incorporating spatial relationships has emerged as an effective approach for establishing natural modes of communication between robots and humans. As per the research conducted by Kuo et al. [50], hierarchical recurrent network coupled with a sampling-based planner can learn and comprehend a series of natural language commands in a continuous configuration space. Similarly, Skubic et al. [51] showcased how a multi-modal robotic interface that utilizes linguistic spatial descriptions along with other spatial information obtained from an evidence grid map can facilitate seamless human–robot dialogue.

Overall, the use of natural language in HRI has shown great potential for improving the ease of communication between humans and robots. The various approaches proposed in the literature demonstrate the diversity of methods that can be employed to address the challenges of natural language understanding in HRI.

### 2.5. User(s) of Interest Detection

To successfully identify UOI in a multi-user environment, the system must determine two crucial pieces of information: the active speaker, who is issuing commands and the intended recipient, whether it be the robot or other users. While active speaker detection (ASD) has received considerable research attention, its application within the context of human–robot interaction (HRI) remains relatively limited. The following subsections delve into noteworthy studies conducted on active speaker detection (ASD) and addressee detection.

#### 2.5.1. Active Speaker Detection (ASD)

The task of identifying the active speaker from a set of candidates in a visual scene, known as *active speaker detection* (**ASD**), is essential for correctly attributing thoughts and ideas to the speaker. In the context of human–robot interaction, ASD can assist in associating commands, requests and suggestions with the appropriate user, whether a robot or a human.

Recent research has focused on developing new techniques and models to improve ASD performance. Pouthier et al. [52] introduced a novel multi-modal fusion scheme based on self-attention and uncertainty to leverage audio and video modalities for ASD. Similarly, Kopuklu et al. [53] proposed a pipeline consisting of audio-visual encoding, inter-speaker modeling and temporal modeling stages, known as ASDNet, for detecting active speakers in challenging environments.

Other approaches have focused on audio-based methods: Kheradiya et al. [54] proposed a technique based on an audio-visual sensor array to localize the active speaker. Chakravarty et al. [55] used audio voice activation detection (AVD) to train a personalized video-based active speaker classifier, modeling the voice of individual users to improve detection accuracy.

Multi-modal approaches have also been explored: Chung et al. [56] minimized audio-video synchronization error to predict active speakers by offsetting the distance between audio and visually embedded features. Roth et al. [57] presented a neural model with a two-stream convolutional neural network for extracting features from audio-visual input followed by a recurrent neural network for active speaker classification.

Additionally, Aubrey et al. [58] presented V-VAD, a method that uses visual information to detect voice activity solely based on the motion vectors obtained from the complex wavelet motion estimation algorithm. However, this approach is limited when the subject’s face suffers from low resolution or occlusion, making it challenging to detect lip contours for feature extraction and classifier design.

Recent work has also explored the use of attention mechanisms and graph convolutional networks to improve ASD accuracy. Tao et al. [59] proposed a feature representation network that captures the long-term temporal context from audio and visual cues and employs cross-attention and self-attention to learn intermodality interactions. Alcazer et al. [60] introduced a novel multi-modal assignation technique based on graph convolutional networks, which simultaneously detects speech events and estimates the active speaker.

In their study, Richter et al. [61] proposed lip movement detection to verify the active speaker and suggested that mutual gaze at the end of an utterance is a significant cue for addressee recognition in multi-party HRI scenarios. Meanwhile, Everingham et al. [62] used the temporal motion of facial landmarks to detect speech, assuming that motion in the lip area indicates speech. Li et al. [63] categorized addressee detection and selection methods for social robots into passive and active methods. Passive methods were designed to detect a predefined visual cue, while active methods utilized human motion, pose, gaze and facial expression to detect the addressee.

#### 2.5.2. Addressee Detection

The primary method used for addressee detection involves detecting eye contact, which focuses on determining whether the gaze is directed towards a specific target.

To sense eye contact in an image, Smith et al. [64] proposed a passive appearance-based approach that relies on gaze locking, rather than gaze tracking, and exploits the unique appearance of direct eye gaze.

Muller et al. [65] introduced a method for detecting eye contact during multi-person interactions, leveraging speaking behavior as weak supervision to train the eye contact detector.

Our approach utilizes facial landmarks to identify the active speaker and simultaneously classify the facing state, i.e., whether the active speaker(s) are addressing the robot or other user(s).

### 2.6. Gaze Estimation

In their study, Mehlmann et al. [66] put forth a modeling approach that leverages gaze for grounding and integrating with the dialog and task management, with a focus on the multi-modal, parallel and bidirectional aspects of gaze. Kompatsiari et al. [67] investigated mutual (social) and neutral (non-social) gaze by conducting experiments involving letter identification with a robot gaze. Their findings suggested that people were more responsive to mutual gaze and were more engaged with the robot when mutual gaze was established.

Wood et al. [68] proposed a model-based approach for binocular gaze estimation using a set of vision algorithms. The approach includes the use of Haar-like feature-based cascade classifiers for detecting eye-pairs and segmenting two coarse regions of interest (ROIs) from the eye-pair region, analyzing the ROIs’ radial derivative to detect Limbus boundary points as the parts of radial edges, and finally RANSAC for robust ellipse fitting. Chen et al. [69] presented a probabilistic eye gaze tracking system that estimates the probability distributions of eye parameters and eye gaze by combining image saliency with the 3D eye model. The system does not require an individual calibration process and can gradually improve when the user is naturally viewing a series of images on the screen. Lu et al. [70] proposed the ALR method, which adaptively selects an optimal set of sparse training samples for gaze estimation via $l_{1}$-optimization. The method integrates subpixel alignment and blink detection into a unified optimization framework to better handle slight head motion and eye blinking in appearance-based gaze estimation.

Sugano et al. [71] utilized a fully calibrated multi-view gaze dataset to generate a large amount of cross-subject training data by performing 3D reconstruction. They trained a random regression forest model on the synthesized dataset to estimate gaze. Liu et al. [72] directly trained a differential convolutional neural model to estimate gaze differences between eye inputs of the same user for a set of sample images. They then compared the inferred differences to a set of subject-specific calibration images to predict gaze direction. Park et al. [73] introduced a deep neural network model that estimates 3D gaze direction from a single-eye input by regressing to an intermediate pictorial representation instead of regressing the pitch and yaw angles of the eyeball. Cheng et al. [74] proposed the FAR-Net, a face-based asymmetric regression network trained with a mechanism that asymmetrically weights and sums the generated loss by the eye gaze directions. The network is optimized by utilizing the eye that can achieve high performance. Park et al. [75] presented the FAZE framework, a novel few-shot adaptive gaze estimation framework that models person-specific gaze networks with ≤9 calibration samples. The framework learns rotation-aware latent representations of gaze via an encoder–decoder architecture.

Mora et al. [76] proposed a multi-modal method that uses depth information to obtain accurate head pose in 3D space, eye-in-head gaze directional information from image data, and a rectification scheme exploiting 3D mesh tracking to facilitate head pose-free eye-in-head gaze direction estimation.

In our approach, we utilized predefined 3D facial points and matched them to a set of extracted estimated 3D facial landmarks of the users from 2D images to infer the gaze direction.

## 3. Methodology

In the upcoming subsections, we will delve into system specifications and the details of how each module was designed and implemented.

### 3.1. System Specification

The proposed framework was implemented on an Ubuntu 20.04 platform equipped with 3.8 GHz Intel R Core(TM) i7-7560U CPU, GTX 1050 GPU and 16 GB system memory. For object detection and pose estimation, a Microsoft Kinect sensor v1 RGB-D camera was employed, while Logitech’s C920 Pro HD webcam was used for other modules. The framework was developed on top of ROS Noetic [77], OpenCV [78], Pytorch [79] and Mediapipe [80].

### 3.2. Object Detection and Pose Estimation

Thanks to deep learning, significant progress has been made in the areas of object classification [81,82,83,84,85,86], detection [87,88,89,90,91,92] and segmentation [93,94,95] from images. However, 3D localization and pose estimation have not progressed at the same pace. One of the main reasons for this is the lack of labeled data, which is not practical to manually infer. As a result, the deep learning community has shifted towards synthetic datasets [96,97,98,99,100] for these applications. Many pose estimation methods utilizing deep learning techniques [101,102,103,104,105] use synthetic datasets for training and have shown satisfactory accuracy.

Although synthetic data offer a potential solution to the problem of insufficient labeled data, generating such data necessitates creating 3D models of photorealistic objects that reflect real-world situations. As a result, generating synthetic data for new objects requires a considerable amount of effort from skilled 3D artists. Furthermore, training and deploying deep learning models demand significant computational resources, making it difficult to achieve real-time object detection and pose estimation on machines with limited computational capabilities. To tackle these problems, we have devised a simplified pipeline that focuses on planar objects, requiring only an RGB image and depth information to accomplish real-time object detection and pose estimation.

In this article, we present an algorithm (Algorithm 1) that leverages a planar pose estimation technique. The underlying assumption in our proposed technique is that the object being considered exhibits an adequate level of texture and can be suitably represented by a planar surface or face. By assuming the presence of texture, we can exploit visual cues and features specific to the object’s surface to facilitate its detection and pose estimation. Additionally, by representing the object as a planar surface or face, we can leverage geometric transformations and related techniques to accurately determine its position and orientation in three-dimensional space.
**Algorithm 1:** Planar Pose Estimation
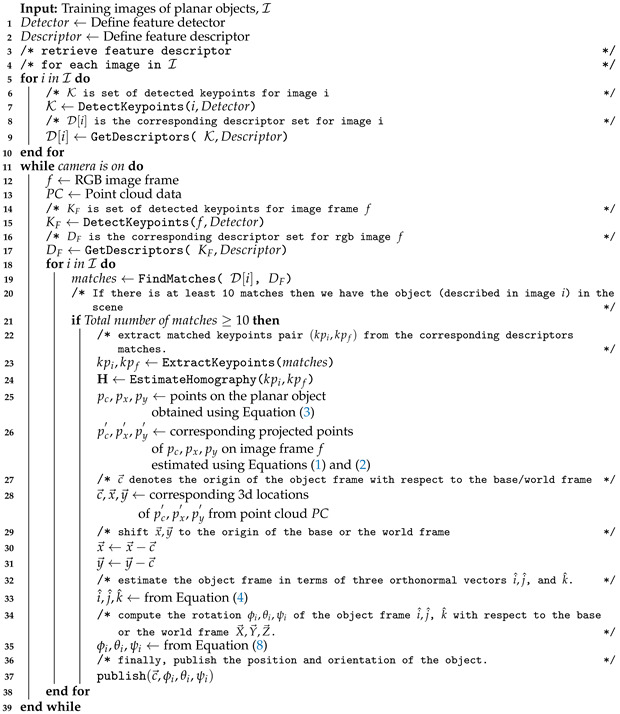


It is important to acknowledge that this assumption may not be universally applicable to all objects in every scenario. Certain objects may lack sufficient texture or possess complex geometries that deviate from a planar representation. Nonetheless, for a significant number of practical cases encountered in human–robot interaction, this assumption holds true and provides a valuable foundation for our technique to achieve reliable and precise results.

The algorithm is composed of four separate stages:

1. Extraction of features from images and finding the corresponding matches.
2. Estimation of homography and performing perspective transformation.
3. Calculation of directional vectors on the surface of the object.
4. Pose estimation of the object from the depth information.

#### 3.2.1. Feature Extraction and Matching

To detect objects in images, our process starts by identifying distinct features within planar objects. These features are patterns in the images that describe their characteristics. We use algorithms to detect features such as edges, corners, interest points, blobs and ridges. Once detected, we transform these features into a vector space using a feature descriptor. This allows us to perform numerical operations on the feature vectors. The feature descriptor encodes the patterns into a set of numerical values, which we can use to compare, match and differentiate one feature from another. By comparing these feature vectors, we can identify similarities in different images, which can aid us in detecting objects. Ideally, the information we gather from these features is invariant to image transformations.

Our research involved the exploration of four descriptors: SIFT [106], SURF [107], AKAZE [108] and BRISK [22]. Although SIFT, SURF and AKAZE are feature detectors and descriptors, BRISK utilizes the FAST [18] algorithm for feature detection.

After extracting and converting the features into vectors, we proceed to compare them in order to determine whether or not an object is present in the scene. In cases where the feature descriptors are non-binary, such as SIFT and SURF, we utilize the nearest neighbor algorithm to find matches. However, as this method becomes computationally expensive in high-dimensional data, and with the addition of more objects, it can adversely impact the real-time pose updating process. To mitigate this issue to some extent, we opted to employ the FLANN [109] implementation of the K-d nearest neighbor search, which is a K-nearest neighbor algorithm approximation, optimized for high dimensional features. In contrast, for binary feature descriptors such as AKAZE and BRISK, we utilized the Hamming distance ratio method to identify matches. If the number of matches exceeds ten, we can infer that the object is present in the scene.

#### 3.2.2. Homography Estimation and Perspective Transformation

**Figure 1:**
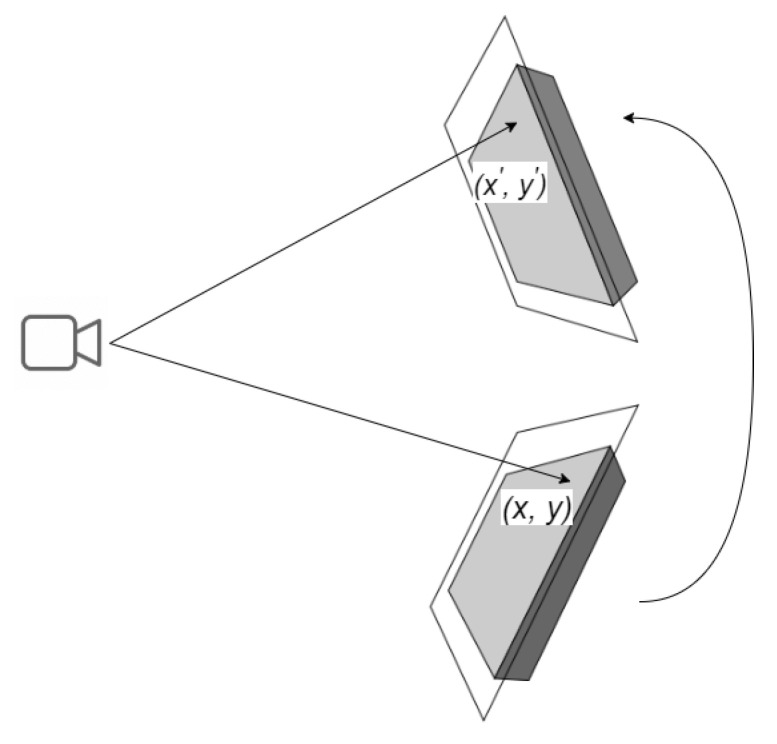
Object in different orientation from the camera.

A homography is a 2D planar projective transformation that can be determined from a given pair of images. It is an invertible mapping of points and lines on the projective plane, as depicted in Figure 1. Essentially, a homography matrix H maps a set of points in one image to their corresponding set of points in another image.

To compute the corresponding points, we can use Equations (1) and (2), which describe the relationship between the projected point $\left(x^{\prime },y^{\prime }\right)$ shown in Figure 1 on the rotated plane and the reference point (x,y).

The 2D point (x,y) in an image can be expressed as a 3D vector (x,y,1), which is referred to as the homogeneous representation of a point on the reference plane or image of the planar object. Equation (1) uses H to denote the homography matrix, while ${\left[x\;y\;1\right]}^{T}$ denotes the homogeneous representation of the reference point (x,y). The projected point $\left(x^{\prime },y^{\prime }\right)$ can be estimated using the values of *a*, *b* and *c* in Equation (2).

(1) $$\begin{array}{c} \left[\begin{array}{c} a\\ b\\ c \end{array}\right]=H\left[\begin{array}{c} x\\ y\\ 1 \end{array}\right]=\left[\begin{array}{ccc} h_{11} & h_{12} & h_{13}\\ h_{21} & h_{22} & h_{23}\\ h_{31} & h_{32} & h_{33} \end{array}\right]\left[\begin{array}{c} x\\ y\\ 1 \end{array}\right] \end{array}$$

(2) $$\begin{array}{c} \left\{\begin{array}{c} x^{{}^{\prime }}=\frac{a}{c}\\ y^{{}^{\prime }}=\frac{b}{c} \end{array}\right. \end{array}$$

To estimate the homography, we utilize the matches obtained from the nearest neighbor search as input. However, these matches can sometimes have false correspondences, which do not correspond to the same real-world feature, thus hindering accurate homography estimation. To overcome this, we employ RANSAC [110], which robustly estimates the homography by only considering inlier matches. RANSAC accomplishes this by attempting to estimate the underlying model parameters and detecting outliers through random sampling, using a minimum number of observations.

Unlike other techniques that utilize as much data as possible to find model parameters and then remove outliers, RANSAC employs the smallest possible set of data points to estimate the model. This approach makes RANSAC faster and more efficient than traditional solutions.

#### 3.2.3. Finding Directional Vectors on the Object

**Figure 2:**
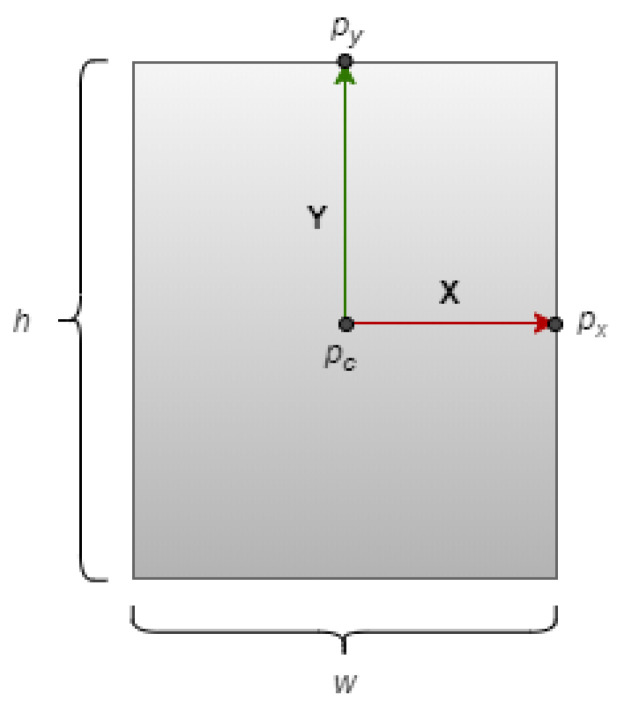
Axis on the reference plane.

In order to determine the pose of a planar object, it is necessary to identify the three orthonormal vectors on the object that describe its coordinate frame. This coordinate frame indicates the object’s orientation relative to the world coordinate system. The first step is to estimate the basis vectors on the planar object, which define the plane, as shown in Figure 2. The basis vector of the 2D plane is computed using Equation (3). The next step involves calculating the cross product of these basis vectors to determine the third directional vector, which represents the surface normal of the object. The object coordinate system is denoted by xyz, while the world coordinate system is represented by XYZ. The object’s orientation axes with respect to its body are defined as follows: 

                                          x→right

                                          y→up

                                          z→towardsthecamera  

Initially, the positions of three points ($p_{c},p_{x},p_{y}$) are extracted from a reference image of a planar object using Equation (3). Subsequently, the corresponding points ($p_{c}^{{}^{\prime }},p_{x}^{{}^{\prime }},p_{y}^{{}^{\prime }}$) are determined from the image obtained from the Microsoft Kinect sensor, utilizing the homography matrix H, as given in Equations (1) and (2).

To obtain the 3D positions of these points, we use point cloud data captured by the Kinect sensor. We denote the positions of $p_{c}^{{}^{\prime }},p_{x}^{{}^{\prime }},p_{y}^{{}^{\prime }}$ as vectors $\vec{c}$, $\vec{x}$ and $\vec{y}$. The vector $\vec{c}$ represents the translation from the object frame to the world frame and the position of the object in the world frame. To align $\vec{x}$ and $\vec{y}$ with the world frame, we center them at the origin of the world frame by subtracting $\vec{c}$.

We calculate the cross product of $\vec{x}$ and $\vec{y}$ to obtain the third axis, $\vec{z}$. However, the estimated axes $\vec{x}$ and $\vec{y}$ may not be perfectly orthogonal due to the homography matrix being an approximation. To resolve this issue, we recalculate the vector $\vec{x}$ by taking the cross product of $\vec{y}$ and $\vec{z}$.

Using these three orthogonal vectors, we calculate the orthonormal unit vectors, $\hat{i}$, $\hat{j}$ and $\hat{k}$, along the $\vec{x}$, $\vec{y}$ and $\vec{z}$ vectors, respectively, using Equation (4). These unit vectors describe the object frame.

**Figure 3:**
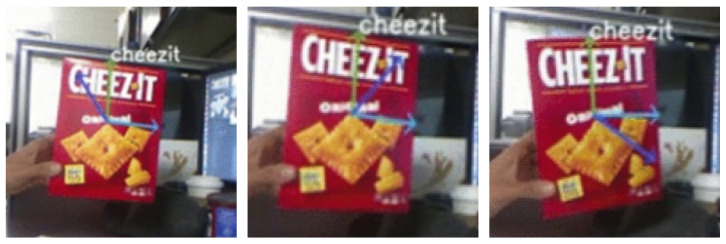
Computed third directional axis projected onto the image plane.

To validate our approach, we project these vectors onto the image plane. The resulting orthogonal axes projected onto the object plane are displayed in Figure 3. This shows that our method provides an accurate estimation of the position and orientation of the planar object in 3D space.

(3) $$\begin{array}{c} \left\{\begin{array}{cc} p_{c} & =(w/2,h/2)\\ p_{x} & =(w,h/2)\\ p_{y} & =(w/2,0) \end{array}\right. \end{array}$$

(4) $$\begin{array}{c} \begin{array}{c} \hat{j}=\frac{\vec{y}}{|\vec{y}|}=\left[j_{X}\;j_{Y}\;j_{Z}\right]\\ \hat{k}=\frac{\vec{x}\times \vec{y}}{|\vec{x}\times \vec{y}|}=\left[k_{X}\;k_{Y}\;k_{Z}\right]\\ \hat{i}=\frac{\vec{y}\times \vec{z}}{|\vec{y}\times \vec{z}|}=\left[i_{X}\;i_{Y}\;i_{Z}\right] \end{array} \end{array}$$

#### 3.2.4. Planar Pose Computation

The orientation of the object relative to a fixed coordinate system is computed using Euler angles. Euler angles comprise three angles that describe the orientation of a rigid body. To obtain the rotation matrix R, which rotates the X axis to $\hat{i}$, the Y axis to $\hat{j}$ and the Z axis to $\hat{k}$, we use Equation (5).

(5) $$\begin{array}{c} R=\left[\begin{array}{ccc} i_{X} & j_{X} & k_{X}\\ i_{Y} & j_{Y} & k_{Y}\\ i_{Z} & j_{Z} & k_{Z} \end{array}\right] \end{array}$$

Euler angles describe the combination of rotations around the X, Y and Z axes, denoted by ϕ, θ and ψ, respectively, as shown in Equation (6). The rotation matrix resulting from these three axis rotations is calculated as the product of three matrices: $R=R_{z}R_{y}R_{x}$ (Equation (7)). Note that the first intrinsic rotation corresponds to the rightmost matrix in the product, while the last intrinsic rotation corresponds to the leftmost matrix.


(6) $$\begin{array}{c} \left\{\begin{array}{cc} R_{x} & =\left[\begin{array}{ccc} 1 & 0 & 0\\ 0 & \cos\phi & -\sin\phi \\ 0 & \sin\phi & \cos\phi \end{array}\right]\\ R_{y} & =\left[\begin{array}{ccc} \cos\theta & 0 & \sin\theta \\ 0 & 1 & 0\\ -\sin\theta & 0 & \cos\theta \end{array}\right]\\ R_{z} & =\left[\begin{array}{ccc} \cos\psi & -\sin\psi & 0\\ \sin\psi & \cos\psi & 0\\ 0 & 0 & 1 \end{array}\right] \end{array}\right. \end{array}$$

(7) $$R=[\begin{array}{ccc} c\theta c\psi & s\phi s\theta c\psi -c\phi s\psi & c\phi s\theta c\psi +s\phi s\psi \\ c\theta s\psi & s\phi s\theta s\psi +c\phi c\psi & c\phi s\theta s\psi -s\phi c\psi \\ -s\theta & s\phi c\theta & c\phi c\theta \end{array}]$$

In Equation (7), *c* and *s* represent cos and sin, respectively.

Solving for ϕ,θ and ψ from (5) and (7), we obtain

(8) $$\begin{array}{c} \left\{\begin{array}{cc} \phi & =\tan^{-1}\left(\frac{j_{Z}}{k_{Z}}\right)\\ \theta & =\tan^{-1}\left(\frac{-i_{Z}}{\sqrt{1-i_{Z}^{2}}}\right)=\sin^{-1}\left(-i_{Z}\right)\\ \psi & =\tan^{-1}\left(\frac{i_{Y}}{i_{X}}\right) \end{array}\right. \end{array}$$

### 3.3. Information Extraction from Verbal Commands

**Figure 4:**
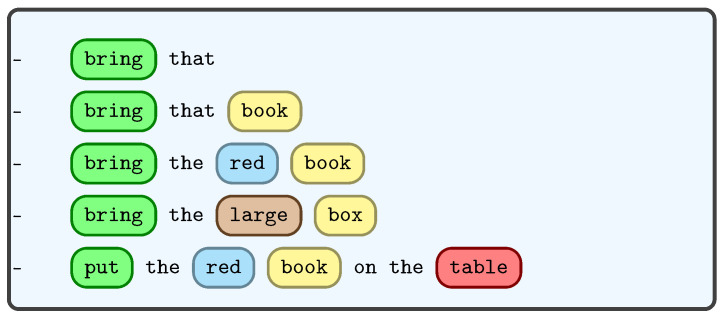
Green box represents the task action; red box indicates the location of the object in the scene; brown box signifies the size of the object; yellow and blue boxes specify the object of interest and the corresponding attributes, respectively.

Verbal communication between humans during collaboration typically involves exchanging specific information such as the task to be performed, the object of interest, navigation directions and the location of interest in the scene. Humans also use descriptive language to clarify the object they are referring to by specifying its general color, pattern, shape, size and relative position, as a means of disambiguation [111]. For example, phrases like “bring that red shirt”, “The book on the left” or “take the small box” define certain task parameters. In our work, we employed a neural network model to extract these task parameters from spoken instructions, as illustrated in Figure 4.

**Figure 5:**
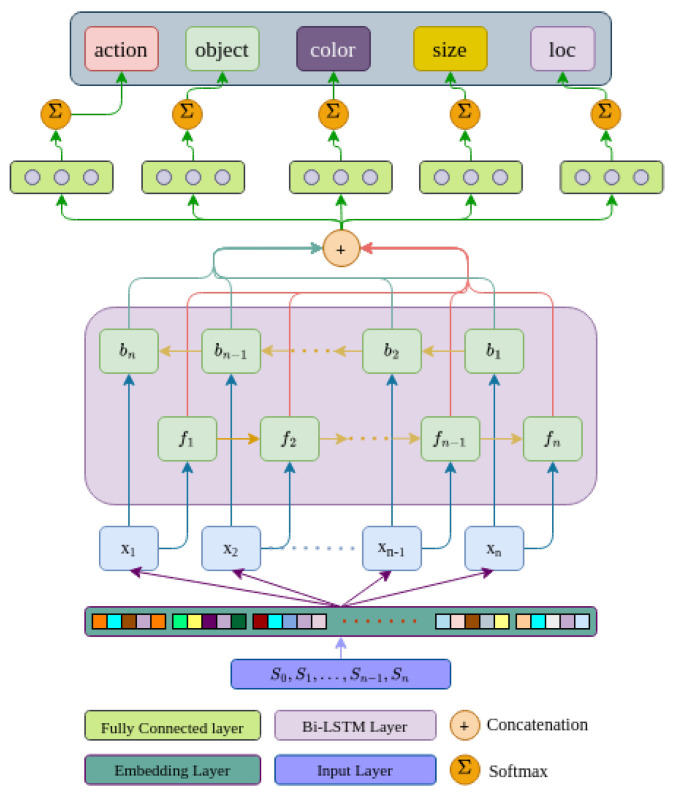
NN model for parameter extraction from verbal commands.

We created a collaborative robotic commands dataset and evaluated 8 architectures to train our model. Ultimately, we determined that a single layer bidirectional long short-term memory (Bi-LSTM) model was the best option. Long short-term memory networks, also known as LSTMs [112], are a type of recurrent neural network (RNN) that have proven to be highly effective in handling sequential data such as text, speech, audio, video and more. In our study, we opted to use a bidirectional LSTM architecture to take advantage of both the past and future contextual information of a given sentence, as well as to learn long-term temporal dependencies while avoiding the problem of exploding or vanishing gradients that are common in traditional RNNs during the backpropagation optimization process. The Bi-LSTM consists of two components, namely the forward LSTM ($f_{i}$) and the backward LSTM ($b_{i}$) as shown in Figure 5.

Our objective was to extract various task parameters from spoken commands, so we developed a deep multi-task learning model. Multi-task learning (MTL) is a machine learning technique where a shared model jointly learns multiple tasks. Deep multi-task learning tries to produce a generalized representation that is powerful enough to be shared across multiple tasks; here, each task denotes a multi-class classification.

**Dataset**: We generated a dataset comprising a large number of commands. Each of these commands consists of action-based instructions and includes essential details regarding one or more of the following parameters: object name, object color, object size and the designated or intended location of the object. In total, the dataset contains an impressive 154,065 samples, each of which is associated with five distinct labels.

**Model Architecture**: The neural network model used in this study comprises three distinct layers: an embedding layer, a bi-directional long short-term memory (Bi-LSTM) layer and a fully connected layer. The vocabulary size of the dataset is denoted by V, and each word is represented by a one-hot encoding of dimension $W\in R^{1\times V}$. The input data in the form of sequences or sentences comprise *n* words and are passed through the embedding layer E.

Embeddings can transform categorical or discrete variables into learned, low-dimensional continuous vectors. Neural network embeddings have the ability to reduce the dimensionality of categorical variables and represent them in the transformed space. The embedding layer $E\in R^{V\times d}$, where d≪V represents the lower-dimensional embedding vector, which is then fed into the Bi-LSTM layer.

After the forward and backward LSTMs have been processed, their outputs are concatenated and fed into four fully connected (FCN) layers. Finally, the softmax activation function is applied to classify the four task parameters. We utilized the cross entropy loss function (Lc) to measure the performance of each classifier, and we then computed the mean of these losses ($Lm=\frac{1}{5}\Sigma_{c=1}^{5}L_{c}$) to update our model. Our approach ensures that the model can effectively classify the input data into one of the five task parameters.

### 3.4. User(s) of Interest Detection

Research has shown that the facing and the gaze transition pattern of the speaker and listener have a strong association with turn-talking [113,114,115]. Vertegaal et al. [116] reported a high probability of the interacting parties facing each other during speaking and listening. Motivated by these findings, we decoupled the problem into two subproblems:

- Detecting the facing state—determining whether the user is facing the camera (robot’s viewpoint) or not.
- Detecting the speaking state—determining whether the user is speaking or not.

This separation results in four possible states: (*i*) speaking and facing, (*ii*) speaking but not facing, (*iii*) not speaking but facing, and (*iv*) not speaking and not facing. If the user is in state *i*, we can infer that they are verbally communicating with or addressing the robot and therefore consider them as a user of interest (UOI).

To address these subproblems, we utilized a third-party library to extract facial landmarks and generated a dataset of these landmarks for both the facing and not-facing states of users.

#### 3.4.1. Dataset

We generated a dataset containing facial landmarks for each participating member in different collaborating scenarios. We leveraged Google’s Mediapipe [80] library to extract facial landmarks for each of these scenario configurations.

**Figure 6:**
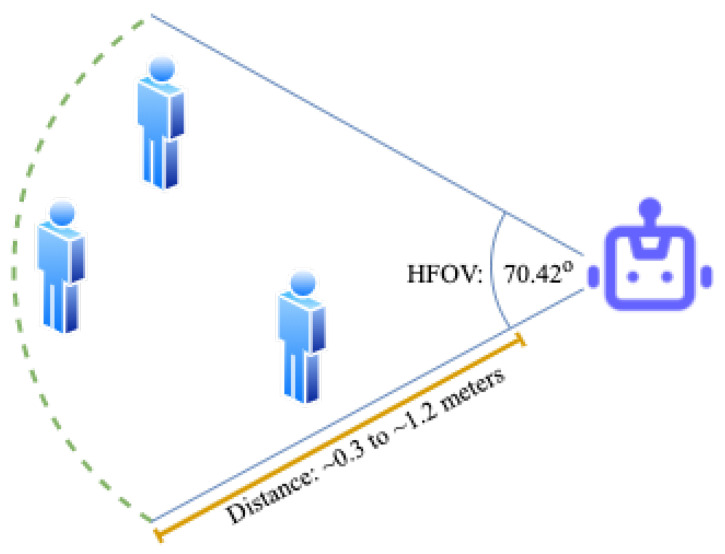
Positioning of the users.

**Data Acquisition**: Logitech’s C920 Pro HD webcam was used which has a horizontal field of view (HFOV) of 70.42${}^{\circ }$. Mediapipe’s face mesh module could consistently extract facial landmarks at a max distance of 1.2 m. Hence, the users were positioned at different distances ranging from 0.3 to 1.2 m and at different angles within the HFOV (see Figure 6).

We defined four states for the participating users:

- Facing the camera, not speaking;
- Facing the camera, speaking;
- Not facing the camera, not speaking;
- Not facing the camera, speaking.

Each dependable frame, from which the system was able to extract facial landmarks, produced 468 3D landmarks per user that were categorized as either facing or not facing, and speaking or not speaking. The dataset, denoted as $D\in R^{S\times U\times 468\times 3}$, contains *S* samples and *U* users in the scene. To ensure consistency across the samples, the data are mean-shifted and undergoes mean-max scaling.

#### 3.4.2. Data Analysis

**Figure 7:**
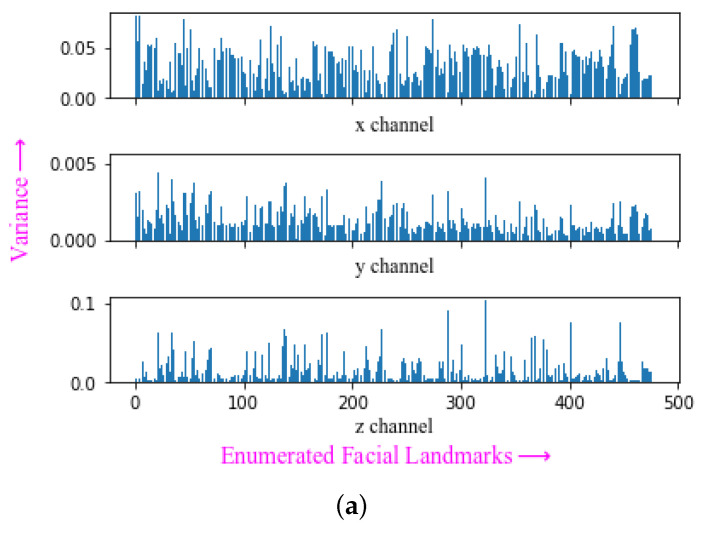

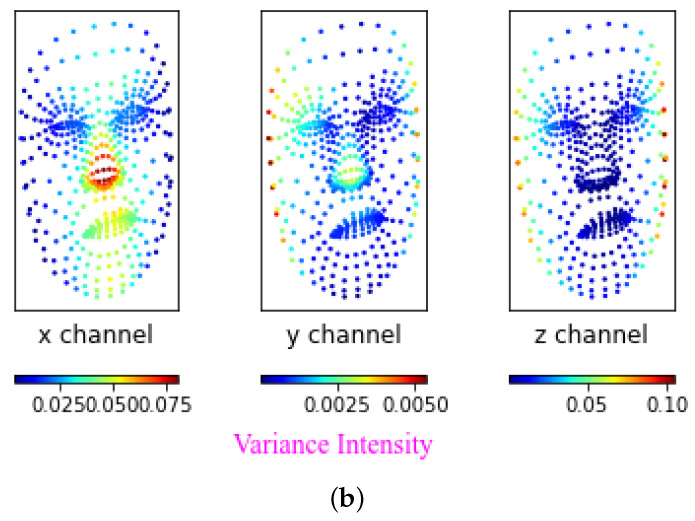
Information contained by the facial landmarks: (**a**) Variance across the enumerated facial landmarks; and (**b**) Variance intensity of the facial landmarks projected onto the face.

The face landmark model [117] detects the face landmarks, each containing 3 values representing the *x*, *y* and an estimated *z* position in the frame. The *x* and *y* coordinates are normalized screen coordinates, while the *z* coordinate is scaled as the *x* coordinate under the weak perspective projection camera model [118]. Figure 7a illustrates the variance across the 468 facial landmarks, while Figure 7b demonstrates the different variance intensities for different facial landmark position. This provides some insight into the information contained across different channels and landmarks. Channel *x* has the most variance for the facial landmarks around the nose and mouth, for channel *y* around the nose and the left and right edges of the face and for channel *z* left and right edges of the face.

**Figure 8:**
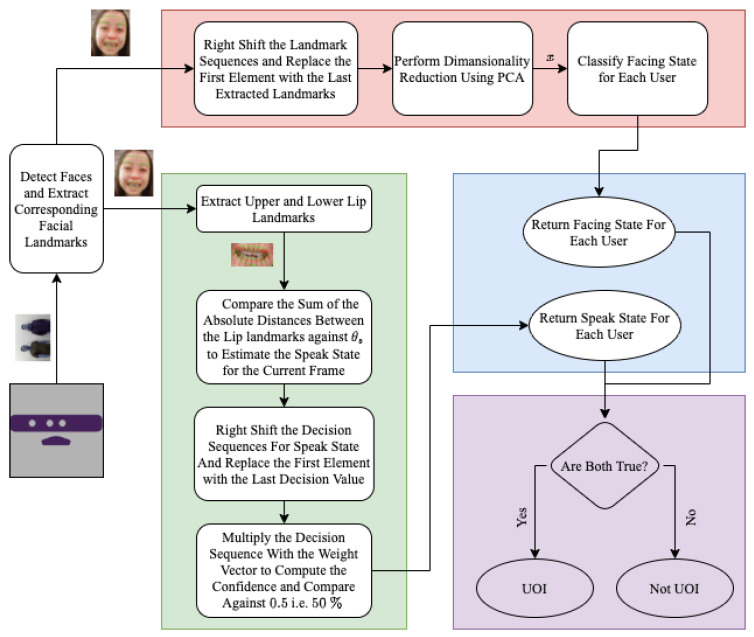
System overview for resolving UOI.

The proposed approach is composed of two distinct stages, illustrated in Figure 8. The first stage involves identifying whether the user is facing the camera, while the second stage involves determining whether the user is speaking or not. The face landmark model [117] is employed to extract facial landmarks from the users in the scene, which are subsequently utilized by two concurrent processes for facing state detection and speaking state estimation. The output from both processes is then combined to ascertain whether the users in the scene are UOIs or not, as outlined in Algorithm 2. The proposed framework’s architecture is described in detail in the following sections.
**Algorithm 2:** Resolving User of Interest by Estimating Facing and Speaking State
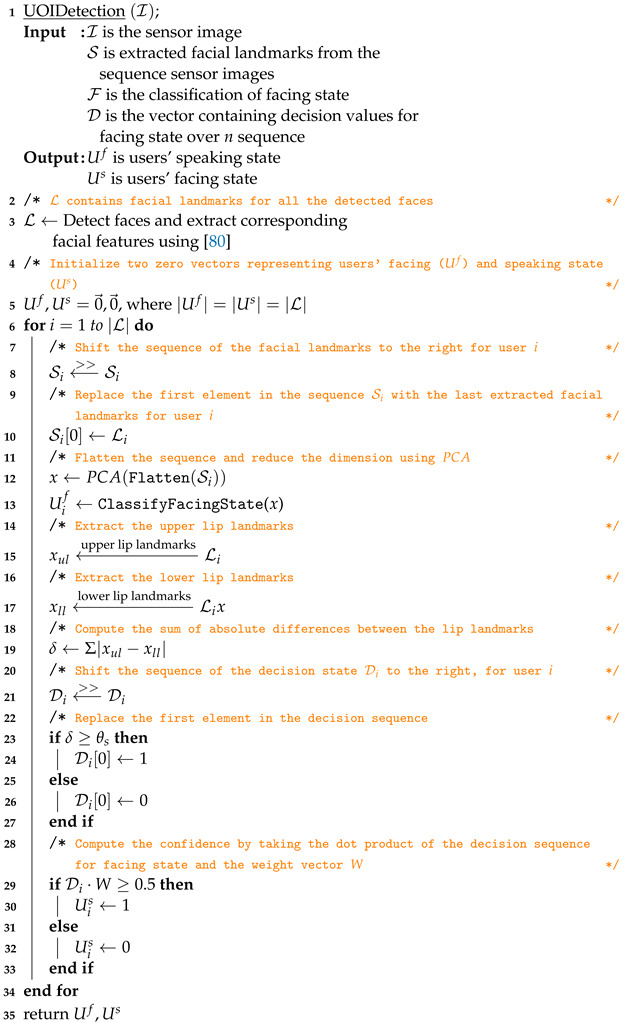


#### 3.4.3. Detecting the Facing State

The system first tries to estimate whether the user is looking towards the robot. To achieve this, principal component analysis (PCA) is employed on the dataset D to reduce its dimensions while preserving 95% of the information. The resulting transformed dataset, D*pca*, is divided into training set D*train* and testing set D_*test*_ at a ratio of 70% to 30%, respectively.

In our study, we trained the data using six widely recognized and publicly available machine learning algorithms. These algorithms were selected based on their widespread usage and proven effectiveness across diverse domains. By evaluating the performance of these algorithms, we aimed to assess their effectiveness and suitability for our specific research objectives. The following algorithms were included in our training process:

1. Nearest neighbors;
2. Gaussian process;
3. Decision tree;
4. Random forest;
5. AdaBoost;
6. Naive Bayes.

#### 3.4.4. Detecting the Speaker

The facial landmarks on the upper and lower lips were selected to further analyze and detect the lip motion for active speaker detection. The assumption was that, when a user speaks, the distance between their upper and lower lips increases. The absolute distance between the corresponding lip points was calculated and scaled using a min–max scaler. This distance was then compared to a threshold value, $\theta_{s}$, to determine whether the user was speaking. However, when pronouncing bilabial consonants such as “b” or “p”, the lips touch, causing the system to detect a non-speaking state. To address this issue, the decision of 1 for speaking and 0 for not speaking was stored in a decision vector, $D_{s}$, of length *n*. A normalized weight vector, $W_{s}$ (as shown in Equation (9)), was then applied to the decision vector to mitigate these types of noise. Each frame was given more significance than the previous one, so the decision for the most recent frame was weighted more heavily. We used a normalized logarithmic growth function for generating this weighted voting scheme.

(9) $$W_{s}=\frac{A}{\sum_{i=1}^{n}A_{i}}$$

where:
$\;A=\langle y|\forall x\in K,y=base^{x}\rangle ;base=2$K=〈k∣∀j∈{0,1,2,..,n−1},k=α+j∗δ〉$\;\delta =\frac{1-\alpha }{n}$α=0.1

In Equation (9), *A* denotes the weight values which are then normalized by dividing with the summation of the vector.

The decision vector is multiplied element-wise by the weight vector to compute the confidence of the decision for that frame and is compared against $\theta_{d}=0.5$, i.e., 50% to resolve the speaking state.

### 3.5. Pointing Gesture Recognition

**Figure 9:**
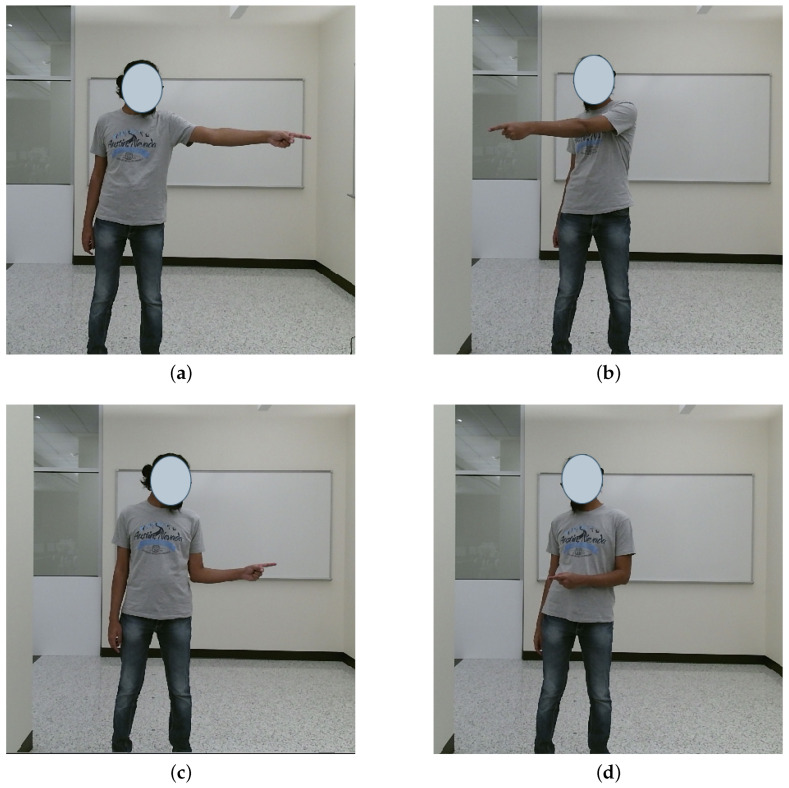
Gesture categories: (**a**) Extended outward; (**b**) Extended across; (**c**) Bent outward; and (**d**) Bent across.

**Figure 10:**
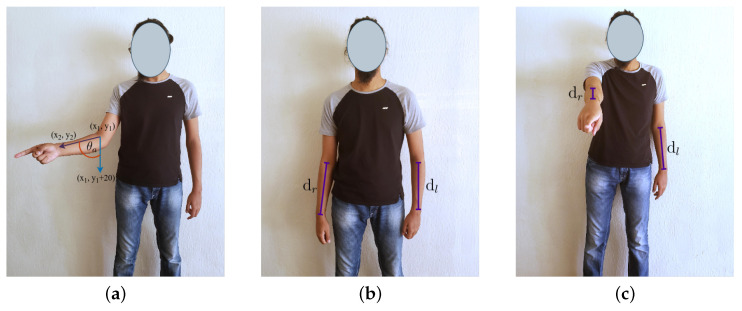
Measures for different pointing states. (**a**) Generated angle $\theta_{a}$; (**b**) Length of forearms $d_{l},d_{r}$ when not pointing; and (**c**) Straight.

To predict the pointing gestures and general pointing direction, we utilized AlphaPose [119] to extract skeletal joint locations. We made the assumption that the user would use only one hand at a time for pointing, for simplicity. Pointing gestures were categorized by Park et al. [43] as either large or small, which we labeled as extended (Figure 9a,b) and bent (Figure 9c,d) arm gestures. In addition, the relative direction of the forearm with respect to the body during the pointing gesture was classified into three categories: across (Figure 9b,d), outward (Figure 9a,c) and straight (Figure 10b).

In order to detect pointing gestures, we utilize the angle $\theta_{a}$, as illustrated in Figure 10a, which represents the angle between the forearm and a vertical line. If this angle is less than a predetermined threshold value $\theta_{t}$, we may infer that a pointing gesture has been performed. Conversely, if the forearm angle is smaller when compared to the angle formed when the user is pointing (as shown in Figure 10b), it may be concluded that the user is not performing a pointing gesture. In the scenario where the user points directly towards the camera (as depicted in Figure 10c), the forearm angle approaches 0, and to address this, we measure the ratio of forearm lengths $\rho_{a}$. When a pointing gesture is not detected, the lengths of the identified forearms should be equivalent (as demonstrated in Figure 10c). However, if there is a notable disparity between the lengths of the forearms, it may be inferred that the user is executing a pointing gesture towards the camera (or its proximity) using the corresponding arm (as shown in Figure 10b). In addition, we determine the pointing direction *d* by analyzing the relative positions of the wrist and the elbow of the pointing arm, which may be used to augment navigational commands.

#### 3.5.1. $\theta_{a}$ Calculation from Wrist and Elbow Location

We only required the locations of certain joints from the extracted skeletal joints, namely the left elbow, left wrist, right elbow and right wrist. Therefore, our method will still function properly even if certain body parts are obscured, as long as the joints in the pointing hand are detected. Let us denote the skeletal joint coordinates of the elbow as $\left(x_{1},y_{1}\right)$ and the wrist as $\left(x_{2},y_{2}\right)$. We can define the pointing 2D vector centered at the origin as $\vec{a}=\left(x_{2}-x_{1},y_{2}-y_{1}\right)$. We will compare this vector with a vertical vector, denoted as $\vec{v}=\left(0,1\right)$. The pointing angle, $\theta_{a}$, can be calculated using Equation (10).

(10) $$\theta_{a}=\cos^{-1}\frac{\vec{a}\;\cdot \;\vec{v}}{\left|a||vs.\right|}$$

In order to determine whether the forearm is performing a pointing gesture, we compare the angle of the forearm, $\theta_{a}$, to a threshold angle, $\theta_{t}$. If $\theta_{a}$ is greater than $\theta_{t}$, we then examine the *x* coordinates of the wrist and elbow to identify the general direction of the pointing gesture (left or right, relative to the body). To determine whether the user is pointing straight ahead, we compare the ratio of the length of the forearm of interest to the length of the other arm, denoted as $\rho_{a}$, to a predefined ratio, $\rho_{t}$. We used a value of 0.8 for $\rho_{t}$ and a threshold angle of $15^{\circ }$ for $\theta_{t}$.

#### 3.5.2. OOI Estimation from Pointing Gesture

For each detected object, the bounding box can be defined as a list of four 2D line segments $BB=[s_{1},s_{2},s_{3},s_{4}]$; $s_{i}$ is defined by the following parametric equation:

(11) $$s_{i}=\left(a_{i},tb_{i}\right)=\left(V_{i},\left\{\begin{array}{cc} t(V_{i+1}-V_{i}) & \mathrm{if}\;i<4,\\ t(V_{1}-V_{i}) & \mathrm{else} \end{array}\right.\right)$$

**Figure 11:**
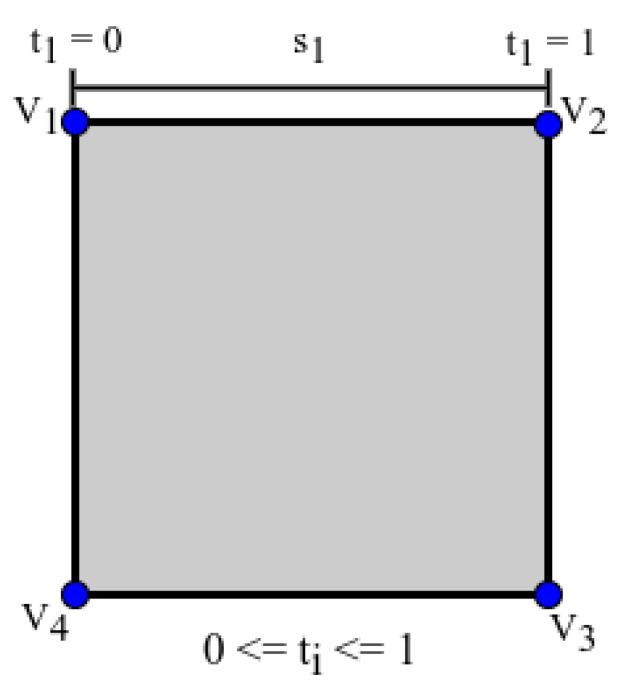
Visualization of the parametric equation of a segment.

Here, $V_{i}$ refers to the $i^{th}$ vertex of a quadrangle bounding box (1≤i≤4) with $0\leq t_{i}\leq 1$ indicating the position of a point on a segment. When $t_{i}=0$, it indicates the initial point, while $t_{i}=1$ represents the final point in the segment. Figure 11 provides a visual representation of this concept. Moreover, the center of each detected object is obtained by averaging the four vertices.

Moreover, to estimate the direction of pointing, a 2D line is computed based on the pixel location of the arm joints, as presented in Equation (12).

(12) $$l_{p}=\left(\left(x_{1},y_{1}\right),t\left(x_{2}-x_{1},y_{2}-y_{1}\right)\right)=\left(a_{p},{\mathrm{tb}}_{p}\right)$$

Here, $l_{p}$ indicates the pointing direction in the image frame, while $\left(x_{1},y_{1}\right)$ and $\left(x_{2},y_{2}\right)$ correspond to the pixel locations of the elbow and the wrist, respectively. The variable *t* represents the position on the line, with −∞<t<+∞.

We can calculate the intersecting point $p_{i}$ for each $s_{i}$ by solving for *t* using Equation (13), where $p_{i}=a_{i}+t_{i}b_{i}$. Then, we measure the distance from the object center to each intersecting point, and determine the minimum distance δ as the object distance. To compute the minimum distance δ for each detected object, Algorithm 3 outlines the necessary steps. The object with the smallest δ is assumed to be the pointed object.

(13) $$t_{i}=\left(a_{p}-a_{i}\right)\times \frac{b_{p}}{b_{i}\times b_{p}}$$

**Algorithm 3:** Minimum Distance Computation Given 2D Pointed Vector and Object Boundary Vertices

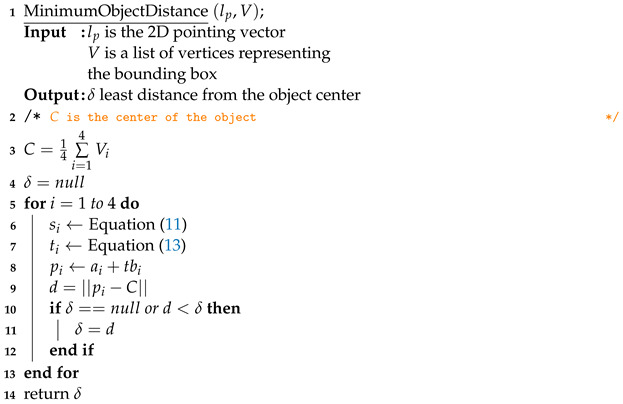



### 3.6. Gaze Estimation

In our work, one of our aims was to estimate gaze based on 2D images. However, due to the nature of gaze estimation being a 3D problem, we took a unique approach. Our initial step involved utilizing a general 3D model (mesh) of a human face, assuming that it would roughly represent the facial proportions of a human face. Subsequently, we leveraged Google’s mediapipe [80] library to estimate the facial landmarks of users from the 2D images. This approach is outlined in Algorithm 4.
**Algorithm 4:** Estimate Gaze Direction from 2D Images
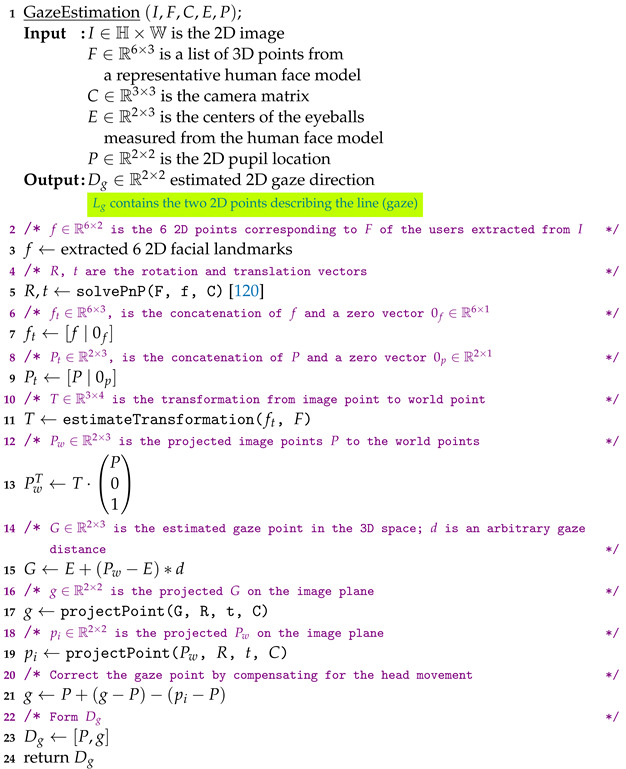


    We chose the tip of the nose as the origin for our coordinate system and identified five other facial landmarks relative to it: the chin, the left corner of the left eye, the right corner of the right eye, the left mouth corner and the right mouth corner. We denote this set of points as $p_{o}\in R^{6\times 3}$. We obtained six 2D points $p_{i}\in R^{6\times 2}$ from the mediapipe [80], and the corresponding estimated 3D points in world coordinates as $p_{e}\in R^{6\times 3}$. Using the pinhole camera model, we computed the 3D to 2D and 2D to 3D transformations.

The pinhole camera model is a mathematical model (Equation (14)) that describes the relationship between points in the 3D world and their projection to the 2D image plane. Using this equation, we can obtain a transformation to project a 3D point into the 2D image plane.

The distortion-free projective transformation given by a pinhole camera model is shown below.

(14) $$sp=A\left[R|t\right]P_{w}$$

where $P_{w}={\left[X_{w}\;Y_{w}\;Z_{w}\right]}^{T}$ is a 3D point expressed with respect to the world coordinate system, $p={\left[uvs.1\right]}^{T}$ is a 2D pixel in the image plane, *A* is the camera intrinsic matrix, *R* and *t* are the rotation and translation that describe the change in coordinates from world to camera coordinate systems (or camera frame) and s is the projective transformation’s arbitrary scaling and not part of the camera model.

**Figure 12:**
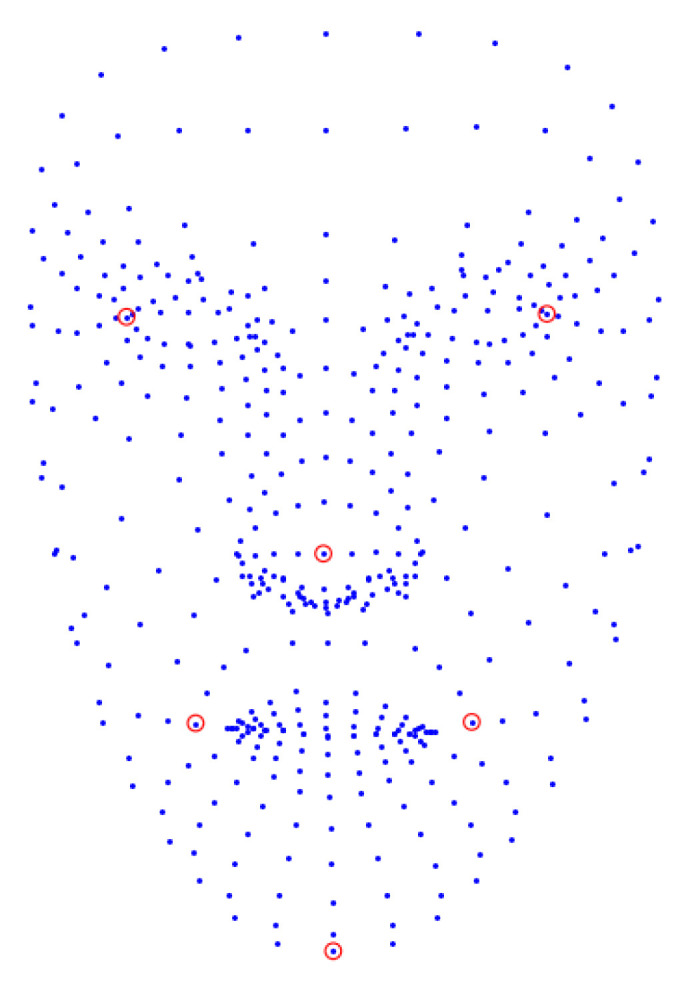
Selected facial landmarks.

The rotation (*R*) and translation (*t*) vectors are computed from the 6 2D image points selected from the extracted estimated facial landmarks (Figure 12) and the corresponding predefined 3D model points; the selected 6 2D points are circled in red in Figure 12. The open source implementation of the [120] is used to calculate the transformation matrix which is applied to project the 3D world points onto the 2D image plane; this provides us with some indication/notion of where the estimated gaze direction points to in the 3D space.

Next, we start by taking the 2D image coordinates (x,y) of the pupil and converting them into 3D model coordinates by appending a 0 to create the tuple (x,y,0). We apply an affine transformation to this tuple using a transformation matrix to project it into 3D space. Next, we utilize the obtained 3D model points of the pupils and the predefined eye center points to determine the gaze direction. To find the intersection point of the gaze with the image plane, we utilize Equation (15) and solve for *s*.

(15) s=c+(p−c)t

where:
$\;s\in R^{1\times 3}$ is the intersection point of the gaze and the image plane$\;p\in R^{1\times 3}$ is the predefined pupil location$\;c\in R^{1\times 3}$ is the predefined eye center location$\;t\in R^{1}$ is the distance between the subject and the camera

It is important to take into account the rotation of the head to ensure that our method for estimating gaze is not affected by any movement of the head. To achieve this, we rely on the estimated initial position of the head to calculate the corrected gaze direction using Equation (16).

(16) $$g=l_{p}+\left(g_{i}-l_{p}\right)-\left(h_{p}-l_{p}\right)$$

where:
$\;g\in R^{1\times 2}$ is the corrected gaze point on the image plane;$\;l_{p}\in R^{1\times 2}$ is the estimated location of the left pupil on the image plane;$\;g_{i}\in R^{1\times 2}$ is the projected gaze point on the 2D plane;$\;h_{p}\in R^{1\times 2}$ is the projected head pose on the image plane.

In order to address unexpected variations or incorrect estimations, a weighted voting mechanism is employed across *n* frames to mitigate the impact of inaccurate estimations. This involves applying a normalized weight vector $W_{s}$ to the estimated gaze direction over *n* vectors (estimated gaze direction). As each subsequent frame holds more significance than the preceding one, the estimation from the most recent frame carries greater weight than earlier ones. To generate this weighted voting scheme, we used a normalized logarithmic growth function, as shown in Equation (17).

(17) $$W_{s}=\frac{A}{\sum_{i=1}^{n}A_{i}}$$

where:
$A=\langle y|\forall x\in K,y=base^{x}\rangle ;base=2$K=〈k∣∀j∈{0,1,2,..,n−1},k=α+j∗δ〉$\delta =\frac{1-\alpha }{n}$α=0.1

In Equation (17), *A* denotes the weight values which are then normalized by dividing with the summation of the vector.

## 4. Results and Discussion

In the upcoming subsections, we will review the experiments and discuss the outcomes for each individual module.

### 4.1. Object Detection and Pose Estimation

We evaluated the proposed algorithm by comparing the accuracy of object recognition, pose estimation and execution time of four different feature descriptors.

**Figure 13:**
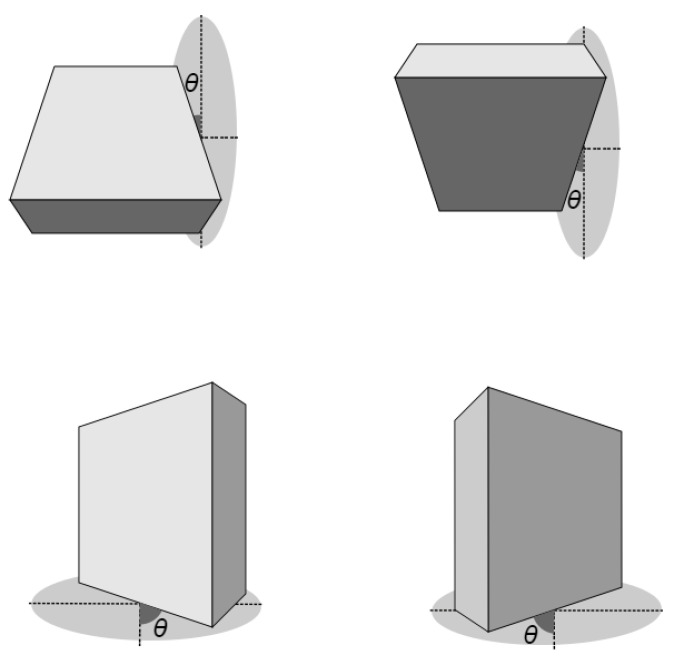
Out of plane rotation.

In order to achieve accurate homography estimation, it is necessary for the system to have sufficient observable features. Otherwise, good matches may not be found, resulting in failure. As a result, our object detection and pose estimation method place a constraint on the out-of-plane rotation θ, as depicted in Figure 13. Specifically, if the object’s out-of-plane rotation exceeds θ, it cannot be recognized by the system. In addition, for real-time applications, fast execution is crucial to enable multiple object detection and pose estimation. We tested four different descriptors on various planar objects, and the comparative results are presented in Table 1. The execution time was measured for the object detection and pose estimation step. AKAZE and BRISK had shorter processing times for detection and pose estimation, which would result in a higher frame rate. However, SIFT and SURF offered greater out-of-plane rotational freedom.

To evaluate the accuracy of homography estimation for planar objects under increasing out-of-plane rotations, we compared the difference in *RMS* (ϵ, as shown in Equation (18)) between the re-calculated $\vec{x}$ and the original $\vec{x}$ (denoted as ${\vec{x}}^{{}^{\prime }}$ in the equation). If the homography estimation is accurate and assuming that the depth information is reliable, then the original and recalculated $\vec{x}$ should be (almost) same. Thus, the ϵ gives us an indication of how much the estimations are off.

In order to estimate the out-of-plane rotation, our approach involved several steps. Firstly, we positioned the object on an angular scale Figure 13 and centered it within the image frame. This alignment was achieved by matching the corner distance between the object and the image frame, and ensuring that the object’s center coincided with the center of the image frame. Then, we rotated the object, and the horizontal out-of-plane angular rotation was measured by referencing the angular scale. The same thing was performed for the vertical out-of-plane rotation by rotating the camera by $90^{\circ }$.

**Figure 14:**
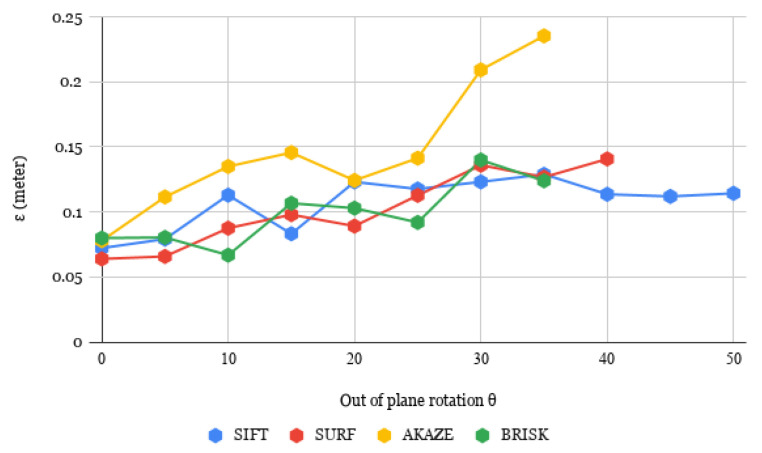
Out of plane rotation vs. ϵ.

The two estimated vectors $\vec{x}$ and $\vec{y}$, which represent the basis of the plane of the planar object, are ideally orthogonal to each other, but this is rarely the case in practical settings. The values of ϵ presented in Figure 14 provide an average measure of the error in homography estimation for different out-of-plane rotations. As depicted in Figure 14, AKAZE produced significantly higher ϵ values compared to the other methods, indicating a larger error in homography estimation. On the other hand, the remaining methods showed comparable ϵ values within a close range.

To evaluate how the execution time for object detection scales up with an increasing number of objects, we opted to use SIFT and SURF. Table 2 displays the mean processing time for object detection, indicating that, in all cases, SURF took approximately 50% longer than SIFT for detection. Based on this finding and previous results, we selected SIFT for subsequent experiments.

**Figure 15:**
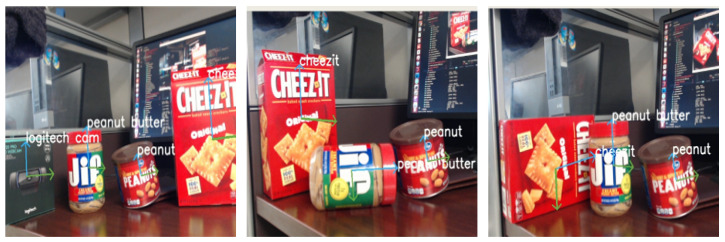
Multiple object detection with estimated planar vectors.

The system demonstrated the ability to detect multiple objects in real-time while simultaneously estimating their corresponding poses. Figure 15 depicts the detected objects with their estimated directional planar vectors. The system also exhibited robustness to in-plane rotation and partial occlusion.

**Figure 16:**
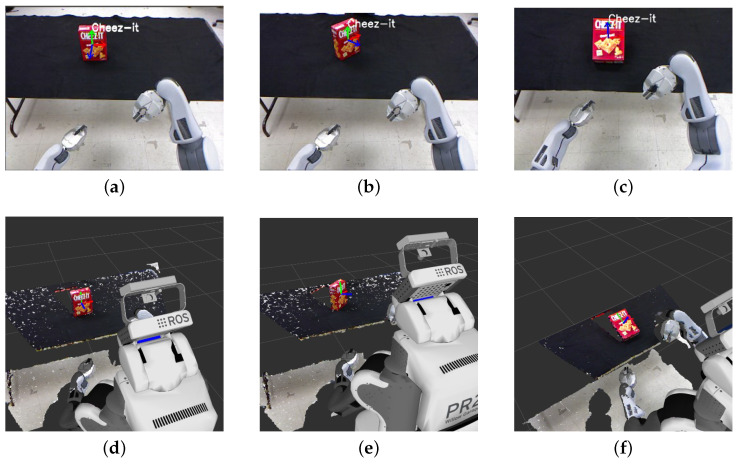
(**a**–**c**) are recovered poses from the robot’s camera and (**d**–**f**) are corresponding poses visualized in RViz.

**Figure 17:**
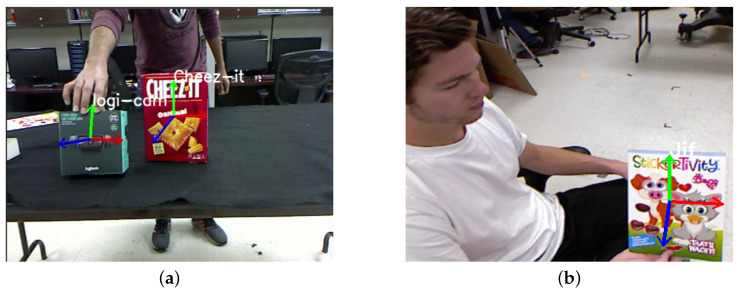
Pose estimation performance: (**a**) Pose estimation of multiple objects; and (**b**) Estimated pose of an object held by a human.

To validate the accuracy of the pose estimation, we utilized RViz, a 3D visualizer designed for the robot operating system (ROS). The directional axes computed were projected onto the image and the resulting poses were then displayed in RViz. As depicted in Figure 16, we compared the two outputs to qualitatively verify the detection and pose estimation accuracy, which rendered similar results. We extended our experiments to multiple objects, including those held by humans. Figure 17 showcases the concurrent detection and pose estimation of two different boxes and an object grasped by a person.

(18) $$\epsilon =\frac{1}{N}\sum_{i=1}^{N}\left|\right|{\vec{x_{i}}}^{\prime }-\vec{x_{i}}\left|\right|,\;\mathrm{where}\;N\;\mathrm{is}\;\mathrm{the}\;\mathrm{number}\;\mathrm{of}\;\mathrm{frames}$$

### 4.2. Information Extraction from Verbal Commands

**Figure 18:**
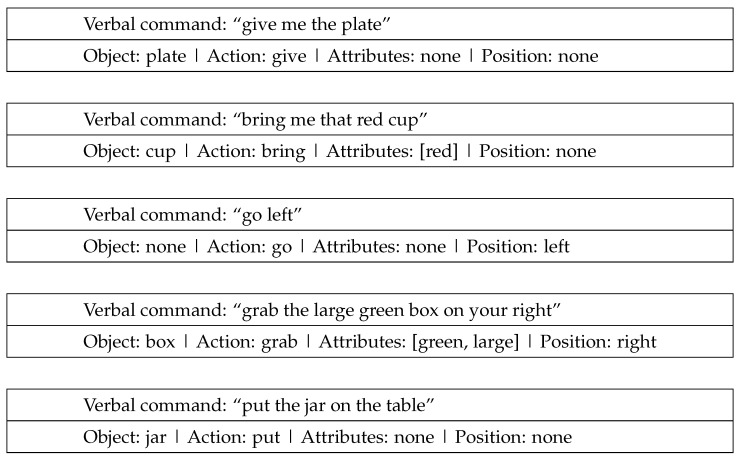
Extracted task parameters from different verbal commands.

**Figure 19:**
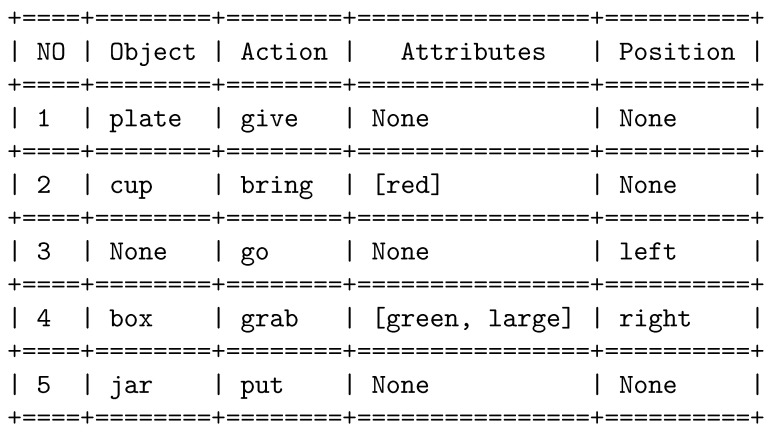
Stored sequential task parameters.

**Figure 20:**
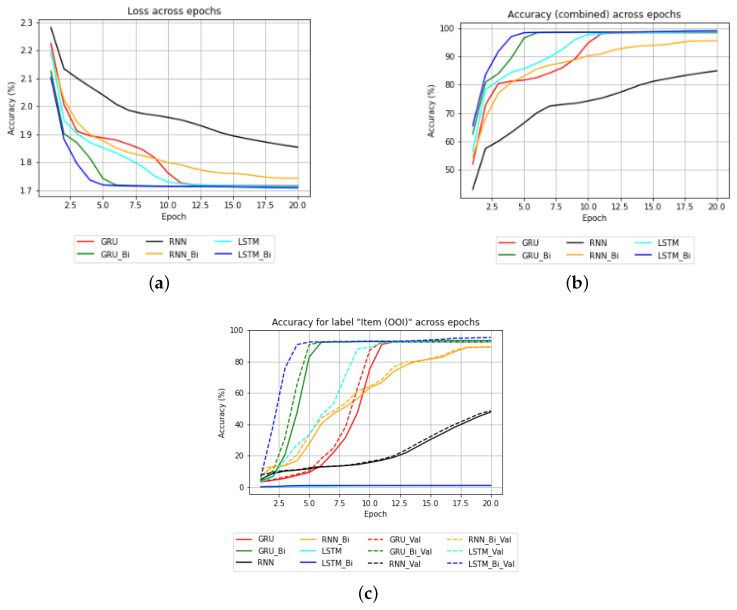
Performance of different models: (**a**) Total loss across epochs; (**b**) Combined accuracy of all the 5 extracted task parameters across epochs; and (**c**) Accuracy for parameter “Item (OOI)” across epochs.

The system receives verbal command and extracts up to five pieces of distinct task information. These parameters are stored so that each task can be executed sequentially. Figure 18 tabulates the received verbal command converted to text and the corresponding extracted task parameters; if no matches are found, the corresponding parameters are set to ***None***. Each command initiates a task and is stored according to the order of task initiation (Figure 19). Additionally, Figure 20 shows the performances of different models. Figure 20a depicts the training loss of each model, Figure 20b shows the combined accuracy and Figure 20c shows the task accuracy for OOI prediction. Table 3 organizes the total number of parameters for the trained models. We can see that, although bidirectional LSTM has more parameters, it has the highest accuracy and converges sooner compared to other models; henceforth, we chose Bi-LSTM as the model for extracting task parameters.

### 4.3. User(s) of Interest Estimation

We conducted a thorough evaluation of our proposed algorithm by assessing its accuracy in recognizing the user of interest (UOI) and detecting speaking states. Additionally, we tested the effectiveness of our approach for active speaker detection by conducting experiments in real-world scenarios.

To conduct these experiments, we instructed participants to interact with each other or with a robot according to predefined instructions. The scenes were designed to include one or two users, and additional users could be added for a wider field of view. For example, in one scenario, participants were instructed to talk to each other, allowing us to confirm that they were speaking but not facing the robot. Similarly, when a participant faced the robot, we assumed that they were the UOI and instructing the robot. We used this information as the ground truth for quantitative evaluation.

Given that our work relies on several modules, we decoupled them to evaluate each module’s performance. These modules include facing and speaking state detection to predict the UOI. Finally, we demonstrated the effectiveness of our framework by showcasing the final results in various interaction scenarios.

#### 4.3.1. Facing State Estimation

We utilized six classifiers to train the data with each sample in the dataset created by concatenating *n* frames. Afterwards, we applied PCA to reduce dimensionality. Table 4 presents a summary of the different classifiers along with their corresponding accuracy and execution time. Among them, the **Gaussian process** demonstrated the highest accuracy, while the second and third best performers were nearest neighbors and AdaBoost, respectively. Although the Gaussian process was comparatively slower in terms of execution time, taking 0.064 ms, it was still fast enough for real-time execution.

#### 4.3.2. Speaking State Estimation

**Figure 21:**
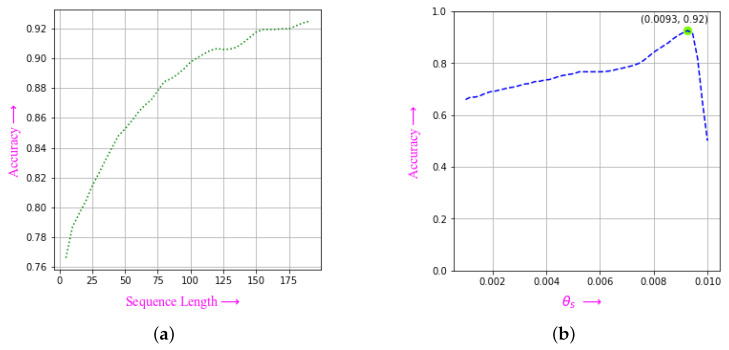
Speaking state estimation performance: (**a**) Accuracy across different sequence lengths; and (**b**) Accuracy across different.

To determine the average accuracies of estimating speaking state for different sequence lengths and $\theta_{s}$ values, a series of experiments were conducted. Each frame in the sequence was evaluated for the speaking state and then multiplied by a weight vector to filter out any noisy decisions. This weight vector is described in the detecting the speaker section. The accuracy results, presented in Figure 21a, clearly demonstrate a positive correlation between accuracy and sequence length, indicating that accuracy increases as the sequence length increases.

As for $\theta_{s}$, the accuracy increased up to $\theta_{s}=0.0093$ and then decreased significantly (as shown in Figure 21b). Based on our findings, a $\theta_{s}$ value of around 0.009 is a good threshold for determining the speaking state.

#### 4.3.3. Final Experiment

**Figure 22:**
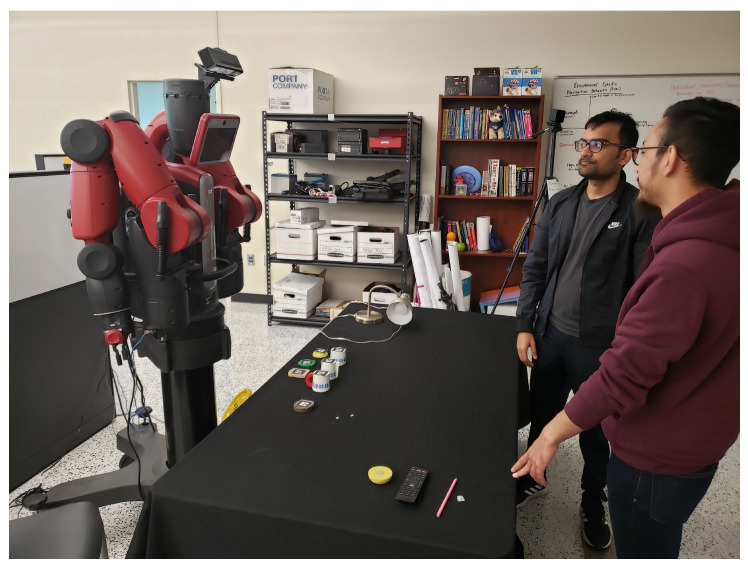
A collaborative multi-party HRI setting.

We conducted experiments involving multi-party interactions, where two users engaged in verbal communication with each other and a robot (represented by a camera). The scenario illustrated in Figure 22 represents a collaborative setting where humans engage in interactions with both objects and a robot. In this particular context, the users have the ability to communicate with one another and the robot through the exchange of instructions. In this depiction, we have a Baxter [121] robot—a humanoid industrial robot equipped with sensors on its head. These sensors enable Baxter to detect the presence of people in its vicinity and provide it with the capability to adjust to its surroundings. The robot plays an active role within this collaborative environment, receiving instructions from the users and executing the corresponding actions to assist in accomplishing the task.

**Figure 23:**
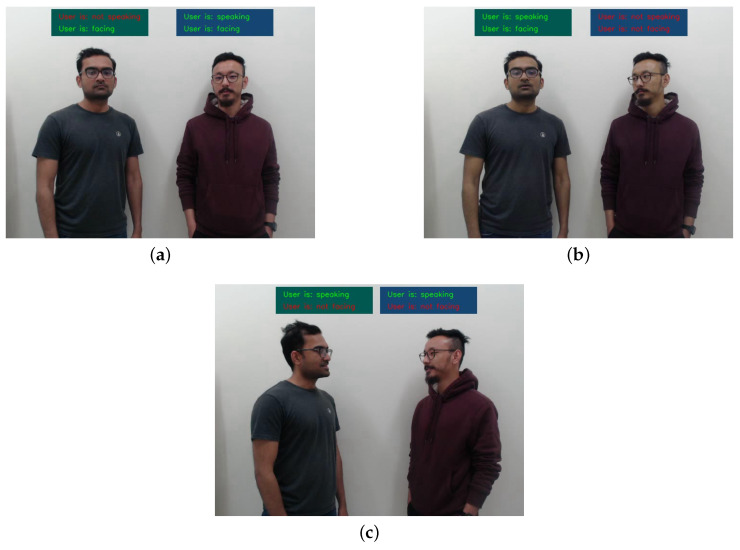
Result for different interacting scenarios: (**a**) Both user facing the robot; (**b**) One of the users facing the robot; and (**c**) Both the users facing each other.

Due to the nature of the collaborative work environment, the humans and the robot are situated in close proximity to one another, ideally within 1.5–4 m. This proximity facilitates efficient communication and interaction, enabling a seamless collaboration. During these experiments, the users faced the addressee while speaking. Three scenarios are depicted in Figure 23. In Figure 23a, both users faced the robot, while the user on the right spoke, indicating that they were addressing the robot and therefore the UOI. In Figure 23b, the user on the right faced the robot and spoke, making them the UOI in this example. In the final example (Figure 23c), both users faced each other and spoke, so there were no UOIs in this scenario. These examples demonstrate the reliable performance of our proposed method in identifying UOIs in multi-party interactive scenarios.

### 4.4. Pointing Gesture Estimation

We conducted experiments in which participants performed a specific pointing gesture in a predefined scenario. The scene consisted of three objects: two books and a Cheez-It box, and the participant could only point to one object at a time. For example, in one scenario, the participant was instructed to extend their right hand and point at the leftmost object, indicating a pointing gesture using their right hand, pointing to the left and targeting the object on the right from their perspective. This information served as the ground truth for quantitative evaluation. The participants were positioned in the center of the image frame and directed to point to different areas of the scene. The pointing direction was classified as “away”, “across” or “straight” for the hand used to point and “not pointing” for the other hand. These experiments were performed with the participant standing at distances of 1.22, 2.44, 3.66 and 4.88 m from the camera.

We evaluated the accuracy, precision and recall of each reliable frame by comparing the prediction to the label. Suppose that a sample frame is labeled “Right hand: pointing; Left hand: not pointing”; in that case, if the prediction is “Right hand: pointing”, we classify the sample as true positive. If the prediction is incorrect and does not detect the pointing right hand, we classify it as false negative. Similarly, if the prediction is “Left hand: pointing”, we classify it as false positive, and if it is incorrect and does not detect the non-pointing left hand, we classify it as true negative.

**Figure 24:**
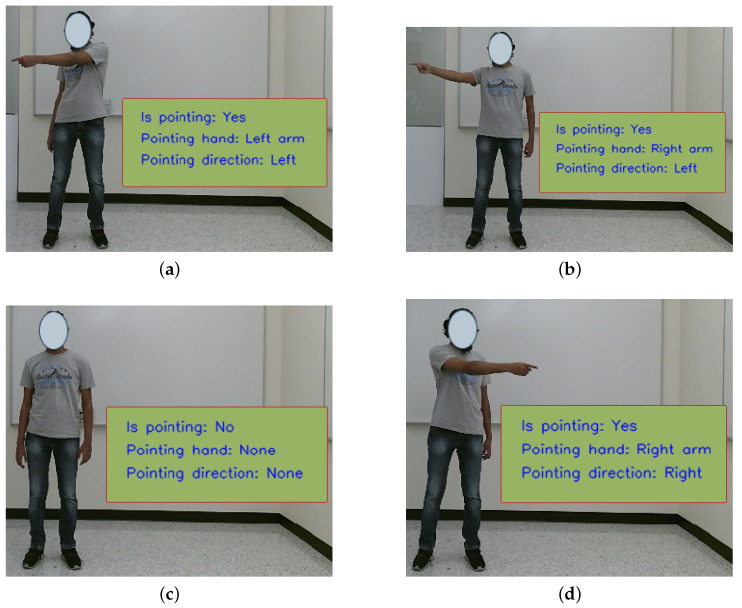
System output with different pointing scenarios: (**a**) Pointing across with left hand; (**b**) Pointing away with right hand; (**c**) No pointing; and (**d**) Pointing across with right hand.

Table 5 presents the accuracy, precision and recall scores for varying distances. Figure 24 provides a visual representation of the system output for different pointing scenarios.

**Figure 25:**
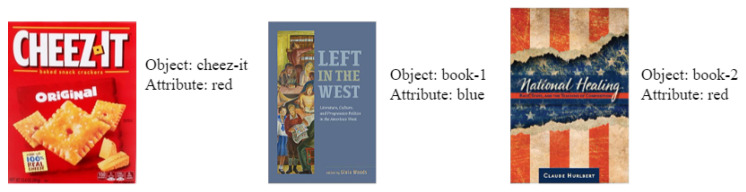
Object attributes.

**Figure 26:**
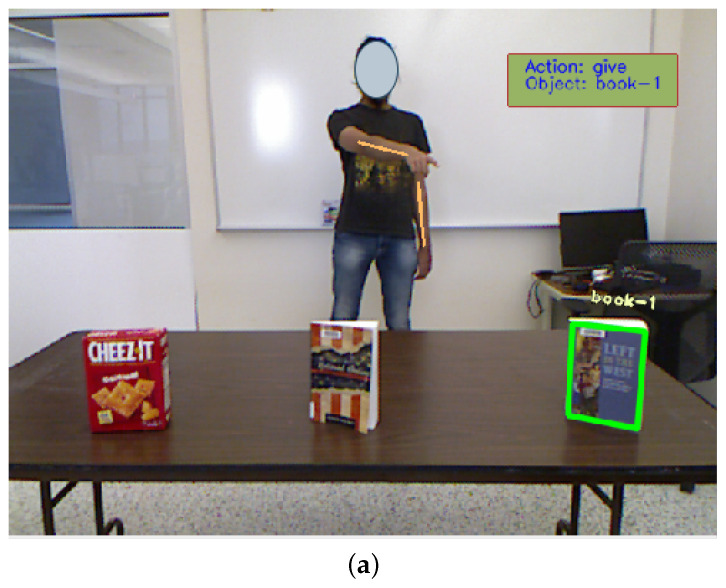

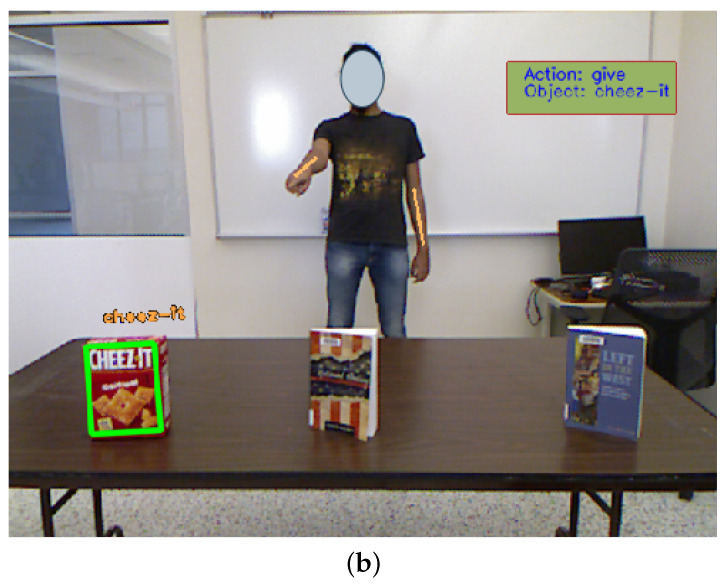
Example scenarios where the user points to different objects while voicing the command “*give me that*”: (**a**) Pointing to the object labeled “**book-1**”; and (**b**) Pointing to the object labeled “**cheez-it**”.

In our experiment, we placed multiple objects with predefined attributes on a table, as shown in Figure 25. The participants were asked to point to a specific object while providing a natural language instruction. Using this information, the system determined the task parameters and provided a follow-up response to address any uncertainties. Figure 26 illustrates two example scenarios, and Table 6 displays the task parameters extracted from each scenario configuration. The “Structured Information” column shows the information extracted from both the pointing state and verbal command. The first column indicates whether the user pointed or not, the second column lists the experiment corresponding to the pointing state and the third column presents different verbal commands with the task action “bring”. The fourth column displays the extracted information from the verbal commands and simultaneous pointing state, while the fifth column lists the predicted object of interest (OOI) requiring the action. The sixth column provides the system’s corresponding response. Ambiguity is indicated by light blue cells.

Ambiguity arises when the intended object of interest (OOI) cannot be inferred from the verbal command and accompanying pointing gesture provided to the system. In such cases, the system is unable to identify the desired object and issues feedback requesting more information, such as “Need additional information to identify object”. Consequently, the system awaits further input from the user, either in the form of a refined command or a clearer pointing gesture. Once these inputs are provided, the system repeats the entire identification process.

Table 6 indicates that, in the “*Not Pointing*” state, ambiguity arises when there is insufficient object attribute(s) (Exp 1, 3) to uniquely identify the **OOI**, leading the system to request additional information. In contrast, in the “*Pointing*” state, ambiguity arises when the pointing direction fails to intersect with any of the object boundaries. Verbal commands can resolve this type of ambiguity by providing additional information. Ambiguity can also occur if the identified objects from the extracted identifiers and the pointing gesture are different. However, the system gives priority to the object inferred from the pointing gesture since the speech-to-text module may sometimes miss transcription.

### 4.5. Gaze Estimation

**Figure 27:**
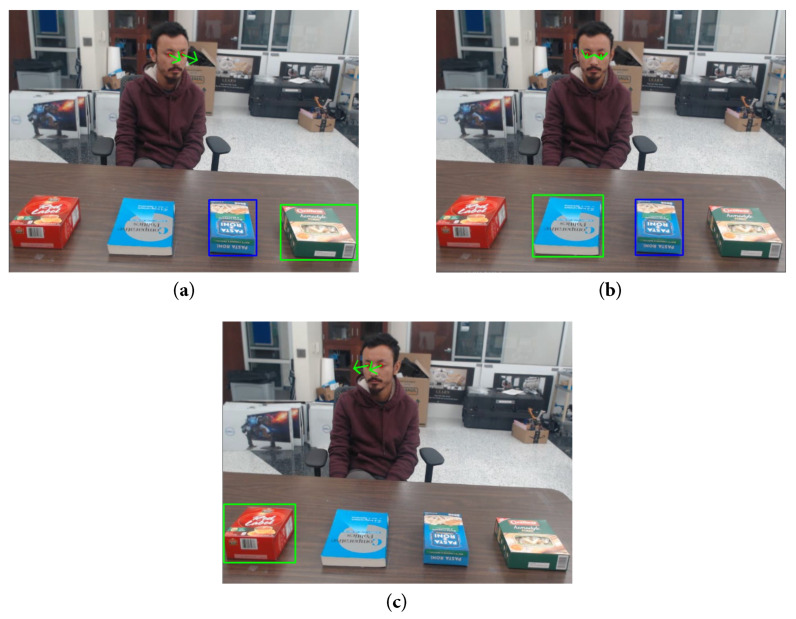
Estimated gaze projected onto the image plane; (**a**) User is looking to their left; (**b**) User is looking straight ahead; and (**c**) User is looking to their right.

We instructed the participants to either look at specific objects within a predetermined setting or give verbal instructions. The environment included four objects, and for instance, in one of the scenarios, the user was directed to look at the leftmost object. Hence, for this specific data sample, we can confirm that the user gazed at the leftmost object (rightmost from the user’s perspective). We regarded this information as the ground truth to evaluate our approach. The gaze estimation system’s final output is shown in Figure 27, where the estimated gaze direction is depicted by green arrows, and the estimated objects of interest (OOI) are represented by bounding boxes. If the gaze distances from multiple objects are less than the threshold distance $\theta_{d}$, the object with the shortest distance is enclosed in a green box, while all other objects are enclosed in blue boxes (Figure 27a,b). This visualization provides insight into the performance of the gaze estimation module.

At the same time, the system is capable of receiving verbal commands and extracting up to five distinct task-related pieces of information. These parameters are then stored in sequence so that each task can be carried out in order. Figure 18 presents the verbal commands that were received and converted into text, along with the corresponding task parameters that were extracted. If no matches were found, the corresponding parameters were set to ***None***. Each command triggered a task and was stored based on the order in which it was initiated. Furthermore, Figure 20 displays the performance of various models. Figure 18a illustrates the training loss of each model, Figure 18b depicts the combined accuracy, and Figure 18c shows the task accuracy for OOI prediction. Table 3 tabulates the total number of parameters for the trained models. It can be observed that, although the bidirectional LSTM has more parameters, it has the highest accuracy and converges more quickly than the other models. As a result, we selected Bi-LSTM as the model for extracting task parameters.

We conducted experiments involving multiple objects placed on a table, each with predefined attributes, while instructing participants to focus on a particular object. Using this information and natural language instructions, the system created task parameters and provided follow-up responses in case of any ambiguities. Table 7 displays sample scenario configurations and the extracted task parameters with the “Structured Information” column, showing information from the pointing state and verbal command. The first column indicates whether the user was looking at an object or not, the second column lists experiments for corresponding pointing states and the third column presents different verbal commands with various task actions. The fourth column shows the extracted information from verbal commands and simultaneous pointing states. Table 8 includes columns containing the identified objects from the corresponding verbal and gaze information, and the final column presents the system’s corresponding response. Yellow cells represent ambiguity or lack of information.

Ambiguity arises when the object of interest (OOI) cannot be accurately inferred from the provided verbal command and gaze cues. In such cases, the system is unable to identify the OOI and, as a result, generates the feedback message "Need additional information to identify object." Thereafter, the system waits for the user to redirect their gaze towards the object and/or modify the command. Once these inputs are received, the system reinitiates the process.

## 5. Conclusions

This paper proposes a multi-modal framework that integrates several modules crucial for an effective human–robot collaborative interaction. The framework encompasses various components such as obtaining the location and pose information of objects in the scene, detecting UOIs and extracting intricate task information from verbal communications. Additionally, the proposed framework incorporates sensor fusion techniques that combine pointing gestures and gaze to facilitate natural and intuitive interactions, providing supplementary or disambiguated task information that might be unclear or missing. The integration of multi-modal and sensor fusion techniques enhances the framework’s ability to facilitate human–robot interaction in complex and dynamic environments, enabling seamless collaboration and achieving desired outcomes.

To identify the objects present in the scene, we employed a feature-detector-descriptor approach for detection and a homography-based technique for pose estimation. Our approach uses depth information to estimate the pose of the object in a 2D planar representation in 3D space. SIFT was found to be the best feature-detector-descriptor for object recognition, and RANSAC was employed to estimate the homography. The system could detect multiple objects and estimate their pose in real-time.

Verbal communication serves as a means to extract detailed information pertaining to a given task, such as commands for actions and descriptions of objects. This process is complemented by the recognition of pointing gestures, whereby the general direction towards a pointed object is estimated in order to facilitate a natural interaction interface for the user. The resulting information is organized into specific categories, known as parameters, which can be analyzed and used to generate a structured command that can be easily interpreted by a robot.

Next, we presented a technique for the real-time detection of the user of interest (UOI) in multi-party verbal interactions in a collaborative human–robot interaction (HRI) setting. The approach involves estimating whether the user is facing the robot (or camera) and determining the active speaker(s) using a dataset of facial landmarks extracted from participants interacting in predefined settings. Machine learning algorithms were trained to determine the best model for estimating the facing state, with Gaussian process having the highest accuracy. The distance between the landmarks on the lips was used to determine the active speaker. The framework was evaluated in multi-party interaction scenarios, and the results demonstrated the effectiveness of the approach in determining the UOIs.

We introduced an approach for recognizing pointing gestures in a 2D image frame, which can detect the gesture and estimate both the direction of the pointing and the object being pointed to in the scene.

To infer the gaze direction, the gaze estimation module matches a set of extracted estimated 3D facial landmarks of the user from 2D images to predefined 3D facial points.

The gesture detection and gaze estimation modules are then combined with the verbal instruction component of the framework and experiments are conducted to assess the performance of these interaction interfaces.

We also explored task configuration formation by presenting various natural language instructions, interaction states and the recorded task parameters in a table. Additionally, the information is compiled into named parameters and analyzed to create a structured command for robotic entities. If any parameter is missing or unclear, the system provides feedback.

The system can be further improved by extracting particular information and patterns from the user dialogues; this would help in determining UOI even when the user is not facing the robot. Additionally, incorporating reliable 3D information would improve precision and eliminate ambiguity. Furthermore, investigating more complex interaction scenarios with multiple users and intricate dialogues could lead to the development of more meaningful HRI systems.

## Figures and Tables

**Table 1:** Comparison of feature descriptors.

Descriptor	Maximum out of Plane Rotation (degree)	Execution Time (s)
SIFT	$48^{\circ }\pm 2^{\circ }$	0.21 s
SURF	$37^{\circ }\pm 2^{\circ }$	0.27 s
AKAZE	$18^{\circ }\pm 1^{\circ }$	0.05 s
BRISK	$22^{\circ }\pm 2^{\circ }$	0.06 s

**Table 2:** Execution time of SIFT and SURF for multiple object detection.

Number of Objects	Detection Time (s)	
	SIFT	SURF
1	0.06 s	0.09 s
2	0.11 s	0.17 s
3	0.17 s	0.26 s
4	0.22 s	0.35 s
5	0.28 s	0.45 s
6	0.34 s	0.54 s

**Table 3:** Number of model parameters.

Model	Total Parameters
GRU	41,371
GRU_Bi	59,963
RNN	32,795
RNN_Bi	42,811
LSTM	45,659
LSTM_Bi	68,539

**Table 4:** Performance of Different Classifiers.

Classifier	Accuracy (%)	Execution Time (ms)
Nearest neighbors	93.88	0.012904
Gaussian processi	**98.70**	0.064134
Decision tree	84.73	0.000025
Random forest	82.29	0.000111
AdaBoost	91.69	0.002113
Naive Bayes	70.82	0.000272

**Table 5:** Pointing gesture recognition.

Distance	Accuracy	Precision	Recall
4.88	1	1	1
3.66	0.995	1	0.99
2.44	0.995	1	0.99
1.22	0.995	1	0.99

**Table 6:** Generated task parameters.

Pointing State	Exp#	Verbal Command	Structured Information	Identified Object	Feedback
*Pointing*	1	bring that, bring me that	*{action: “bring”, pointing_identifier: True, object: “book”,* *object_identifiers: {attributes: null, position: null}}*	“book-1”	None
	2	bring the red book	*{action: “bring”, pointing_identifier: True, object: “book”,* *object_identifiers: {attributes: “red”, position: }}*	“book-2”	None
	3	bring that red thing	*{action: “bring”, pointing_identifier: True,* *object: null, object_identifiers: {attributes: “red”, position: }}*	“cheez-it”	None
*Not pointing*	1	bring that, bring me that	*{action: “bring”, pointing_identifier: False,* *object: null, object_identifiers: {attributes: null, position: null}}*	None (ambiguous)	"Need additional information to identify object"
	2	bring the red book	*{action: “bring”, pointing_identifier: False,* *object: “book”, object_identifiers: {attributes: “red”, position: null}}*	“book-2”	None
	3	bring that red thing	*{action: “bring”, pointing_identifier: False,* *object: null, object_identifiers: {attributes: “red”, position: “right”}}*	None (ambiguous)	”Need additional information to identify object”

**Table 7:** Extracted Task Parameters from Verbal Command.

Gazing State	Exp#	Verbal Command	Structured Information
Looking at an object	1	bring me that	action: bring, object: none, identifier: none, location: none
	2	could you give me that blue thing	action: give, object: none, identifier: red, location: none
	3	give me that small box	action: give, object: box, identifier: small, location: none
	4	put the blue box on the table	action: give, object: box, identifier: blue, location:Table
Not looking at any object	1	bring me that	action: bring, object: none, identifier: none, location: none
	2	could you give me that blue thing	action: give, object: none, identifier: red, location: none
	3	give me that small box	action: give, object: box, identifier: small, location: none
	4	put the blue box on the table	action: give, object: box, identifier: blue, location: table

**Table 8:** OOI Estimation and Feedback.

Gazing State	Exp#	Identified Object from Verbal Info	Object Detected from Gazing Info	Feedback
Looking at an object	1	None (not enough info)	Object 3 (Pasta Roni)	None
	2	None (ambiguous)	Object 2 (Book)	None
	3	Object 3 (tea box)	Object 3 (tea box), object 2 (book)	None
	4	Object 3 (Pasta Roni)	Object 3 (Pasta Roni)	None
Not looking at any object	1	None (not enough info)	None (ambiguous)	“Need additional information to identify object”
	2	None (ambiguous)	None (ambiguous)	“Need additional information to identify object”
	3	Object 3 (tea box)	None	None
	4	Object 3 (Pasta Roni)	None	None

## Data Availability

The data that support the findings of this study are available on request from the corresponding author. The data are not publicly available due to privacy.

## Abbreviations

The following abbreviations are used in this manuscript:

UOI	User(s) of interest
OOI	Object(s) of interest
